# From Antimicrobial Activity to Topical Translation: An Integrated Framework for Testing, Cytotoxicity Assessment and Formulation of Plant Extracts, Essential Oils and Honey

**DOI:** 10.3390/pharmaceutics18091111

**Published:** 2026-09-03

**Authors:** Diana Constanța Pelea, Laura Maria Endres, Laura Maghiar, Teodor-Andrei Maghiar, Mădălin Florin Ganea, Csaba Nagy, Corina Moisa, Gabriela Ciavoi, Georgiana Ioana Potra Cicalau, Olimpia-Daniela Frenț, Mariana Ganea

**Affiliations:** 1 Preclinic Department, Faculty of Medicine and Pharmacy, University of Oradea, Universității Str., No. 1, 410087 Oradea, Romania; diana_pelea@uoradea.ro; 2 Department of Psycho-Neurosciences and Rehabilitation, Faculty of Medicine and Pharmacy, University of Oradea, Universității Str., No. 1, 410087 Oradea, Romania; laura_endres@yahoo.com; 3 Department of Dermatovenerology, Clinical County Emergency Hospital Bihor, Gheorghe Doja Str., No. 65, 410169 Oradea, Romania; 4 Department of Surgical Disciplines, Faculty of Medicine and Pharmacy, University of Oradea, Universității Str., No. 1, 410087 Oradea, Romania; 5 Department of Surgery, Pelican Hospital, Corneliu Coposu Str., No. 2, 410450 Oradea, Romania; 6 Faculty of Medicine and Pharmacy, University of Oradea, 1 Decembrie Square, No. 10, 410028 Oradea, Romania; ganea.madalinflorin@student.uoradea.ro; 7 Doctoral School of Biomedical Sciences, University of Oradea, Universității Str., No. 1, 410073 Oradea, Romania; nagy.csaba@student.uoradea.ro; 8 Department of Pharmacy, Faculty of Medicine and Pharmacy, University of Oradea, Nicolae Jiga Str., No. 29, 410028 Oradea, Romania; corinamoisa@hotmail.com (C.M.); ofrent@uoradea.ro (O.-D.F.); mganea@uoradea.ro (M.G.); 9 Department of Dental Medicine, Faculty of Medicine and Pharmacy, University of Oradea, 1 Decembrie Square, No. 10, 410073 Oradea, Romania; gciavoi@uoradea.ro (G.C.); cicalau.georgiana@uoradea.ro (G.I.P.C.)

**Keywords:** natural antimicrobial products, plant extracts, essential oils, medicinal honey, antimicrobial susceptibility testing, selectivity index, topical formulation, biofilm

## Abstract

**Background/Objectives:** Antimicrobial resistance has renewed interest in plant extracts, essential oils and honey as topical adjuvants, yet antimicrobial potency, host-tissue safety and formulation performance are usually reported in separate bodies of literature, and the material tested is often characterized only superficially. This structured narrative review aimed to integrate these dimensions into a single decision-oriented pathway. **Methods:** The Web of Science Core Collection was searched using seventeen predefined strings across fourteen thematic domains, restricted to English-language articles and reviews from 2021 to 2026, with supplementary PubMed/MEDLINE searches, hand-searching, and normative documents from the issuing organizations. **Results:** The framework comprises seven decision domains: pre-analytical standardization of each matrix class; antibacterial and antifungal testing with matrix-specific controls; biofilm endpoints named according to what they measure; strain selection tiered from reference to clinical and resistant isolates; host safety from monolayer cytotoxicity to reconstructed human epidermis; selectivity, expressed per microorganism and extended to the resident cutaneous microbiota; and formulation performance, with permeation interpreted against a retention rather than a maximization target. Resistance under sub-inhibitory exposure forms an advanced stage. **Conclusions:** Progression is governed by proposed Go, Conditional Go and No-Go criteria, supported by a matrix-specific control table and a minimum reporting checklist. The framework has not been prospectively validated, and no universal selectivity threshold is proposed.

## 1. Introduction: Antimicrobial Resistance and the Need for an Integrated Approach to Natural Topical Adjuvants

Antimicrobial resistance is a major public health problem with profound implications for the effectiveness of existing anti-infective treatments and for patient safety [1,2]. The growing prevalence of microorganisms resistant to conventional antibiotics has created an acute need for alternative strategies, both for the treatment of infections and for their prevention [3,4,5]. In this context, natural products have regained attention within the scientific community, not as direct substitutes for systemic antibiotics, but as potential adjuvants, particularly for topical applications where high local concentrations can be achieved while limiting systemic exposure and reducing selective pressure at the whole-body level [6].

Plant extracts, essential oils, and honey have been used for centuries in traditional medicine for their antimicrobial, anti-inflammatory, and wound-healing properties [7,8,9,10,11]. Recent advances in analytical chemistry and microbiology have enabled the identification and characterization of the bioactive constituents responsible for these effects, including polyphenols, flavonoids, terpenoids, aldehydes, organic acids, and antimicrobial peptides, and have clarified that natural matrices often act through multiple complementary mechanisms rather than single-target effects [8,9,12,13]. Nevertheless, demonstrating in vitro antimicrobial activity, even when supported by standardized laboratory assays, is not sufficient to justify the development of a safe and effective pharmaceutical product intended for clinical use [3,14,15,16].

A major limitation of the existing literature is the fragmentation of experimental approaches. Many studies focus exclusively on antimicrobial activity, reporting minimum inhibitory concentration (MIC) values without integrating cytotoxicity toward host cells or considering the feasibility of pharmaceutical formulation [3,14,17]. In the absence of such integration, there is a practical risk of promoting products that are microbiologically active yet display an unacceptable safety profile at the concentrations required for efficacy, or that cannot be formulated in a stable and reproducible manner suitable for real-world topical administration [2,6].

Topical applications represent a particularly relevant domain for natural antimicrobial products. Unlike systemic administration, local use permits the attainment of high concentrations at the site of application, while reducing systemic exposure and the likelihood of generalized adverse effects [6]. However, this opportunity is coupled with specific challenges related to cutaneous or mucosal tolerability, the interaction with local microbiota, and variability in absorption and local bioavailability depending on the dosage form and excipient system [6]. For certain natural products, additional issues include physicochemical variability, limited aqueous solubility, volatility, or matrix-driven assay interference, all of which can distort laboratory readouts and complicate translation unless addressed by method adaptation and rigorous controls [3,8,9,16].

Within this context, the need for an integrated approach becomes evident: antimicrobial activity must be assessed together with cellular safety and with pharmaceutical formulation constraints. Such integration enables not only the identification of biologically promising products, but also the rational selection of candidates that remain viable for development after accounting for host–cell tolerability and technological feasibility. A key operational tool for this integration is the Selectivity Index, which links microbial inhibitory potency to host–cell cytotoxic thresholds and supports transparent decision-making early in development pipelines [2,17].

The present article aims to provide a comprehensive and coherent methodological framework for evaluating natural antimicrobial products intended for topical use, emphasizing method standardization, quality control, and transparent decision criteria that can guide selection and formulation. By combining standardized antimicrobial susceptibility testing principles with adaptations appropriate for complex natural matrices, and by aligning antimicrobial endpoints with cytotoxicity assessment and formulation feasibility, the framework is designed to reduce the frequently observed gap between promising laboratory findings and failures encountered in later development stages [1,2,3,15,17,18].

## 2. Literature Search and Framework Development

### 2.1. Review Design and Reporting Approach

This article was developed as a structured narrative review rather than as a systematic review. The aim was not an exhaustive census of published studies, but the construction of a transparent and structured methodological framework at the intersection of three fields of literature that are usually reported in isolation: antimicrobial susceptibility testing of complex natural matrices, cellular and tissue-level safety assessment, and topical pharmaceutical development. The review was informed by the quality domains captured by SANRA, namely explicit justification of the topic, a clearly stated aim, a described literature search, referencing of specific statements, scientific reasoning, and appropriate presentation of data [19]; SANRA is a quality-assessment scale for narrative reviews rather than a reporting guideline equivalent to PRISMA. The search strings and filters reported below are documented reproducibly, although rank-dependent retrieval is date-specific, since both citation ranking and relevance ranking change over time; source selection, by contrast, was purposive and is described as such, and no claim of systematic-review-level completeness is made.

### 2.2. Information Sources

The Web of Science Core Collection was used as the primary bibliographic source and was searched with predefined strings covering all thematic domains of the framework. Targeted supplementary searches were performed in PubMed/MEDLINE for three domains in which the primary search returned insufficient topic-specific literature, namely standardization and authentication of plant material, application of the Selectivity Index, and resistance development under sub-inhibitory exposure. Reference lists of included articles were hand-searched for additional sources, including foundational works published before the main search window. Normative documents were retrieved directly from the issuing organizations rather than from secondary sources; these included the current EUCAST clinical breakpoint tables, the CLSI standards for dilution susceptibility testing of aerobic bacteria (M07) and for performance standards (M100), the CLSI reference methods for antifungal susceptibility testing of yeasts (M27) and filamentous fungi (M38), ISO 20776-1, the OECD test guideline for in vitro skin irritation using reconstructed human epidermis and the OECD guideline on defined approaches for skin sensitisation, the regulatory guidance on quality and performance of topical products, and the ICH guideline on pharmaceutical development [1,2,18,20,21,22,23,24,25,26].

### 2.3. Search Strategy

Searches were run in August 2026 and covered the period from January 2021 to August 2026 for the update of the evidence base, with foundational literature identified separately through hand-searching. Searches were restricted to English-language articles and reviews. Fourteen thematic domains were addressed using seventeen Web of Science search strings, including broad and refined versions where required, covering methodology of antimicrobial testing of natural products; standardization and chemical characterization of plant extracts; chemotype, chemical variability and oxidative status of essential oils; medical-grade honey; biofilm endpoints; antifungal susceptibility testing; cytotoxicity and selectivity; reconstructed skin models, irritation and sensitisation; contact sensitisation to essential oils; the skin microbiome; in vitro release and permeation testing; quality by design and critical quality attributes of topical products; encapsulation and nanostructured delivery of essential oils; and resistance development under sub-inhibitory exposure. Two searches were superseded during development of the strategy and were replaced by refined strings rather than discontinued as thematic domains. T3 combined the term for essential oils with generic terms for chromatographic analysis, oxidation and stability and returned an unmanageably broad result set dominated by routine compositional characterization; it was replaced by T3b, in which those terms were replaced with chemotype, chemical variability, oxidation product, autoxidation, batch-to-batch and standardization. T5 contained a construction error, in that the term wound was placed within the same OR group as the natural-product terms and therefore retrieved records containing no natural product at all; it was replaced by T5b, in which that term was removed from the group, and the endpoint terms were broadened. Both the original and the corrected or refined strings are reported in Supplementary File S1, together with dates and result counts.

Records were exported in full for all non-superseded searches except T1. For T1, only the 1000 most highly cited records of the 1310 identified records were exported. For larger result sets, including T5b, T8, T11b and T12, the complete result set was retrieved in successive batches ranked by citation count. The superseded T3 search was not exported; the 543 records retrieved by the superseded T5 search were retained only as background context and were included in the exported working dataset. A total of 13,825 records were exported into the working dataset and 12,456 remained after de-duplication by digital object identifier and, where absent, by normalized title. The search log and the deduplicated bibliographic working dataset are provided as Supplementary File S2. The three supplementary searches in PubMed/MEDLINE contributed a further 600 screened titles. Between two and seven sources were retained per substantive thematic domain, with one additional source addressing review methodology, yielding 40 peer-reviewed sources from the predefined update searches. The reference list was subsequently verified entry by entry against PubMed, Crossref and publisher records; sources whose metadata could not be confirmed were removed and the corresponding statements re-sourced. These were supplemented by additional relevant literature, including foundational sources identified through hand-searching, and by normative documents retrieved directly from the issuing organizations, yielding 89 references in total. Per-domain counts are reported in Supplementary File S1. Following peer review, search T1 was re-run with identical filters and the complete result set of 1317 identified records was exported in two batches. De-duplication against the working dataset identified 226 records that had not formed part of the original citation-ranked export, of which 124 were published in 2025 or 2026. All were screened by title and abstract against the eligibility criteria; three met them and were incorporated, and the screening record is provided in Supplementary File S2.

### 2.4. Eligibility Criteria and Selection

Records were eligible if they were peer-reviewed articles or reviews relevant to at least one thematic domain and to topical application, and if the methodology was described in sufficient detail to be cited as a source of good practice. Conference abstracts, preprints and non-peer-reviewed sources were excluded, as were studies confined to food applications without transferable methodological content, and articles for which the full text could not be obtained. Priority was given to primary studies for factual statements and to recent reviews for context and synthesis. Normative documents were used only in the version currently in force. Records were screened by title and abstract by one author (D.C.P.), and records retained at that stage were assessed in full text by a second author (L.M.). Disagreements between the two were resolved by a third author (L.M.E.), whose decision was final. Within each thematic domain, sources were retained until the methodological content of that domain was considered covered, rather than against a fixed target number; this accounts for the variation between domains, with standardization of plant material and resistance development requiring seven sources each, while narrower domains were covered by two. Given the narrative design, selection was purposive and prioritized methodological relevance for topical natural antimicrobial products over exhaustive coverage, and the retention decision therefore rests on the judgement of the authors rather than on a predefined quantitative rule. These eligibility criteria were applied to the predefined and targeted evidence searches. Additional peer-reviewed sources identified through hand-searching or contextual supplementation were used selectively to support broader methodological or translational considerations and were not treated as evidence defining the stage-gate criteria.

### 2.5. Framework Construction

The framework was assembled by mapping each retained source onto one of seven sequential decision domains: identity and standardization of the raw material, antimicrobial efficacy under quality-controlled conditions, activity against biofilms, host-tissue safety, selectivity, formulation performance, and translational readiness. Where standardized methods exist, the framework adopts them without modification. Where the physicochemical behaviour of natural matrices makes direct application invalid, the required adaptation is stated explicitly together with the control that preserves interpretability. Where no standard exists, the framework defines a reporting requirement rather than a numerical threshold, so that data remain comparable across laboratories even in the absence of consensus cut-off values. The resulting structure is stage-gated: progression between domains is governed by predefined Go, Conditional Go and No-Go criteria rather than by the accumulation of favourable individual results.

## 3. Rationale for the Use of Plant Extracts, Essential Oils and Honey as Topical Antimicrobial Adjuvants: Biological Foundations and Pharmacological Justification

### 3.1. Antimicrobial Resistance and the Strategic Value of Topical Interventions

The renewed interest in natural products as antimicrobial adjuvants is not the result of a transient scientific trend, but rather a response to well-defined limitations of conventional antimicrobial therapies. Systemic administration of antibiotics, while indispensable in many clinical contexts, is intrinsically associated with broad selective pressure on commensal and pathogenic microbiota, the emergence of multidrug-resistant strains, and a spectrum of adverse effects that may compromise patient compliance and safety [1,2,4]. In contrast, topical applications offer the possibility of localized intervention, allowing high antimicrobial concentrations to be achieved directly at the site of infection while minimizing systemic exposure and reducing the ecological impact on distant microbial communities [6]. It should be noted, however, that topical administration reduces systemic ecological exposure without eliminating local selection pressure at the site of application; sub-inhibitory concentrations necessarily arise at the periphery of the treated area, and the consequences of this are addressed in Section 11.1.

Within this strategic framework, natural products occupy a distinctive position. Their use as topical adjuvants aligns with contemporary antimicrobial stewardship principles, which emphasize targeted, localized, and context-specific interventions rather than indiscriminate systemic exposure [1,6]. Importantly, topical administration does not eliminate the need for rigorous evaluation; rather, it shifts the focus toward local efficacy, tolerability, and formulation-dependent bioavailability, parameters that are often underexplored in studies centered solely on antimicrobial potency.

### 3.2. Plant Extracts as Complex Antimicrobial Systems

Plant extracts are inherently multicomponent systems composed of diverse classes of secondary metabolites, including polyphenols, flavonoids, tannins, alkaloids, and terpenoids, whose relative abundance is influenced by botanical origin, extraction method, and processing conditions [4,7,12]. From a pharmacological perspective, this chemical complexity represents both an opportunity and a challenge. Unlike single-molecule antimicrobials, plant extracts may exert antimicrobial effects through multiple complementary mechanisms, such as disruption of bacterial cell wall integrity, alteration of membrane permeability, inhibition of essential metabolic enzymes, and interference with quorum sensing and other regulatory pathways [5,13,14,27,28].

The simultaneous presence of multiple bioactive constituents may reduce the probability of single-step, target-based resistance, since adaptation to multifactorial stressors is less readily achieved than to single-target agents; this does not, however, exclude adaptation, induced tolerance or efflux-mediated responses under sub-inhibitory exposure, which are documented for several natural matrices and are addressed in Section 11.1 [13,29]. However, this same complexity complicates standardization, reproducibility, and mechanistic interpretation. For topical applications, where reproducible local exposure is critical, variability in extract composition may translate directly into variability in clinical performance unless stringent quality control and characterization are applied [4,30].

### 3.3. Essential Oils: Lipophilicity, Membrane Targeting and Safety Considerations

Essential oils differ fundamentally from aqueous or hydroalcoholic plant extracts in their physicochemical and biological behavior. Their predominant lipophilicity and volatility confer a strong affinity for lipid-rich biological structures, including bacterial cytoplasmic membranes and eukaryotic cell membranes [8,9]. Major constituents such as monoterpenes, sesquiterpenes, and oxygenated derivatives can insert into lipid bilayers, leading to increased membrane permeability, leakage of ions and metabolites, and collapse of the proton motive force [9,31,32].

These mechanisms account for the broad-spectrum antimicrobial activity frequently reported for essential oils and their components, but they also explain their potential cytotoxicity toward host cells [17]. In the context of topical use, this duality necessitates careful dose selection and formulation strategies capable of modulating local exposure. Without such strategies, the concentration range separating antimicrobial efficacy from cellular toxicity may be unacceptably narrow, limiting direct applicability despite promising in vitro results [6,17].

### 3.4. Honey as a Multifactorial Antimicrobial Matrix

Honey represents a unique case among natural antimicrobial products due to its multifactorial mode of action. Its antimicrobial activity arises from the combined effects of high osmolarity, enzymatic production of hydrogen peroxide, low pH, and the presence of phenolic compounds and other bioactive constituents [10,11,33]. Certain honeys also contain specific antibacterial factors that contribute to activity against a broad range of microorganisms, including strains associated with wound infections and biofilm formation [11,33].

From a formulation perspective, honey offers intrinsic advantages, such as high viscosity and natural adherence to tissue surfaces, which can prolong local contact time. However, its composition is highly dependent on botanical source and processing, and uncontrolled variability can significantly influence antimicrobial performance and stability [10,33]. These factors underscore the necessity of rigorous characterization and standardization before integration into pharmaceutical-grade topical products.

### 3.5. Synergistic Local Effects Beyond Antimicrobial Activity

A central argument for the use of natural products as topical antimicrobial adjuvants lies in their ability to combine antimicrobial activity with additional biological effects relevant to tissue repair. Anti-inflammatory, antioxidant, and wound-healing properties have been reported for numerous plant-derived compounds, essential oils, and honey, suggesting potential benefits that extend beyond microbial control [4,12]. In chronic or recurrent cutaneous infections, where persistent inflammation and tissue damage play a major role in pathogenesis, such multimodal effects may contribute significantly to therapeutic outcomes [34,35].

However, these additional effects cannot be assumed a priori and must be evaluated within an integrated experimental framework. Anti-inflammatory or antioxidant activity does not compensate for insufficient antimicrobial efficacy, nor does antimicrobial potency justify neglect of local tolerability. Only through parallel assessment of these dimensions can the true translational value of a natural product be determined [2,6,17].

### 3.6. Positioning Natural Products as Adjuvants Rather than Antibiotic Substitutes

A critical conceptual distinction underpinning this framework is the positioning of natural products as adjuvants rather than direct replacements for systemic antibiotics. In this role, natural products may contribute to reducing local microbial burden, preventing colonization, disrupting biofilms, or enhancing the effectiveness of other therapeutic interventions, without exerting selective pressure comparable to that of systemic antimicrobials [29,36].

This pragmatic positioning aligns with current stewardship strategies and acknowledges both the strengths and limitations of natural products. It also emphasizes the importance of integrated evaluation, in which antimicrobial activity, cytotoxicity, and formulation feasibility are assessed together to support rational development decisions [2,6].

In summary, the rationale for using plant extracts, essential oils, and honey as topical antimicrobial adjuvants is grounded in biological plausibility, pharmacological advantages, and translational opportunity. These advantages can only be realized, however, through rigorous, standardized, and integrated evaluation. The following chapter addresses the general methodological framework required to achieve this integration and to position experimental data appropriately relative to existing standards.

## 4. Positioning the Evaluation of Natural Antimicrobial Products Relative to International Standards

### 4.1. The Need for a Dedicated Methodological Framework for Natural Matrices

The evaluation of natural products with antimicrobial potential requires a methodological framework capable of ensuring reproducibility, comparability, and biological relevance of the data generated. Unlike synthetic antimicrobial agents, which are chemically well-defined and relatively homogeneous, plant extracts, essential oils, and honey are characterized by intrinsic variability arising from biological origin, extraction procedures, processing conditions, and storage stability [3,4,30]. This variability directly affects experimental outcomes and complicates the interpretation of antimicrobial activity if not adequately controlled.

A general framework is therefore necessary to reconcile the rigor of standardized antimicrobial susceptibility testing with the specific constraints imposed by complex natural matrices. Such a framework must preserve the core principles of standardized testing while allowing justified adaptations that maintain methodological validity and translational relevance [1,2,18].

### 4.2. Positioning Natural Product Testing Relative to International Standards

International standards developed for antimicrobial susceptibility testing, including those issued by recognized reference bodies, provide an essential foundation for laboratory evaluation of antimicrobial activity [1,2,18,20]. These standards define critical experimental parameters such as inoculum density, culture media composition, incubation temperature and duration, and criteria for result interpretation. Adherence to these principles is indispensable to avoid systematic bias and to ensure internal consistency.

However, direct and uncritical application of these standards to natural products may be insufficient or even misleading. Standard protocols were designed primarily for pure, water-soluble antimicrobial compounds with predictable physicochemical behavior. Natural products frequently deviate from these assumptions, exhibiting limited solubility, intrinsic color or turbidity, volatility, or interaction with assay materials, all of which can interfere with standard readouts [3,16]. Consequently, positioning relative to standards must be both informed and reflective, recognizing their value while explicitly addressing their limitations.

**Figure 1:**
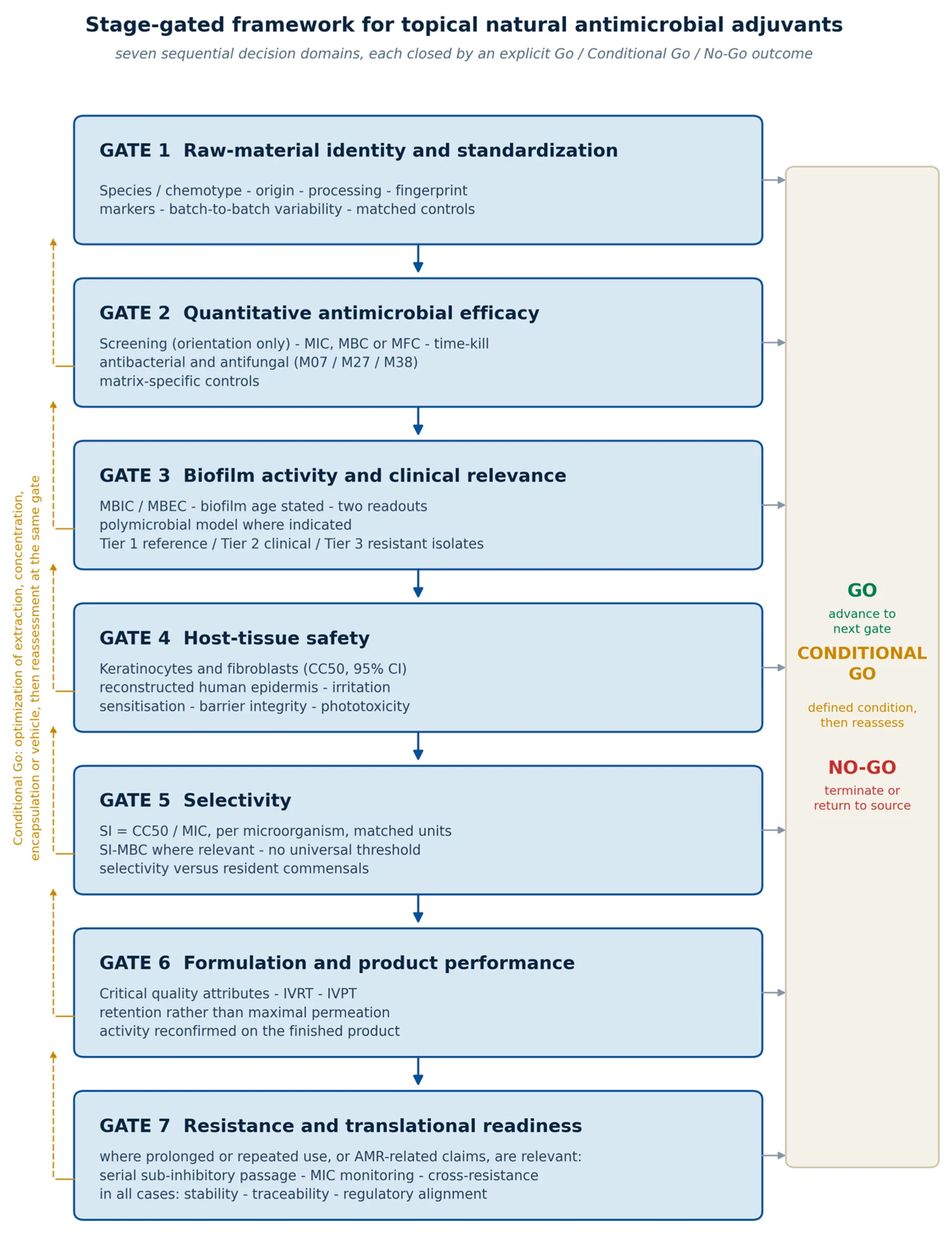
Stage-gated framework for the evaluation and development of topical natural antimicrobial adjuvants. Solid arrows indicate progression to the next gate. The dashed arrow on the left indicates the Conditional Go loop, in which the candidate is reassessed at the same gate after the stated condition has been resolved. The arrows on the right indicate the outcome applicable at each gate.

The overall stepwise evaluation strategy and the associated decision architecture are summarized schematically in Figure 1.

The schematic represents the seven sequential decision domains of the proposed framework. Gate 1 establishes the identity and standardization of the raw material, including species and, for essential oils, chemotype, processing history, chromatographic fingerprint, quantified marker compounds, between-batch variability and the matrix-matched controls that these data define. Gate 2 addresses quantitative antimicrobial efficacy, with screening used for orientation only and minimum inhibitory concentrations and, where relevant, minimum bactericidal or minimum fungicidal concentrations determined under reference conditions. Gate 3 addresses activity against biofilms, with endpoints named according to what they measure, and confirmation across a tiered strain panel progressing from reference strains to contemporary clinical and resistant isolates. Gate 4 covers host-tissue safety, progressing from monolayer cytotoxicity to reconstructed human epidermis and to irritation, sensitisation and barrier endpoints. Gate 5 integrates efficacy and safety through the Selectivity Index, reported per microorganism, and adds selectivity relative to resident commensals. Gate 6 addresses formulation and product performance, including critical quality attributes, in vitro release and permeation testing interpreted against a retention rather than a maximization target, and reconfirmation of activity on the finished product. Gate 7 addresses potential for resistance development under sub-inhibitory exposure and translational readiness.

Each gate is closed by an explicit outcome. A Go outcome permits progression to the next gate. A Conditional Go outcome defines a specific, testable limitation to be resolved, after which the candidate is reassessed at the same gate rather than progressing by default; this iterative loop is indicated on the left. A No-Go outcome terminates development in the current form or returns the candidate to the raw-material stage. The criteria applicable at each gate are detailed in Section 9.8.

## 5. Pre-Analytical Standardization of Natural Products

### 5.1. Knowing What Is Being Tested

A recurrent and underestimated weakness in the literature on natural antimicrobials is that testing frequently begins before the identity and composition of the test material have been established. Antimicrobial and cytotoxicity data acquired on an incompletely characterized matrix cannot be reproduced by another laboratory, cannot be attributed to defined constituents, and cannot support formulation decisions, however rigorous the downstream assays may be [37,38]. Pre-analytical standardization is therefore positioned here as the first stage of the framework rather than as a descriptive preamble, and its outputs are treated as prerequisites for entry into quantitative testing.

The requirement is proportionate rather than absolute. Full metabolomic characterization is neither necessary nor realistic at an exploratory stage. What is required is that the material be traceable, that the analytical identity be documented by at least one orthogonal method, and that between-batch variability be quantified for the constituents held to be responsible for activity [37,39,40]. The minimum information to be recorded for each matrix class is summarized in Table 1. Table 1 constitutes the operational evidence requirement for Gate 1, and the matched controls it defines are those applied throughout Section 6 and Section 7.

### 5.2. Plant Extracts: Botanical Identity and Extraction Traceability

For plant extracts, botanical authentication is the first and most frequently omitted requirement. Species identity should be confirmed by an appropriate method and supported by a deposited voucher specimen, since morphological identification alone is unreliable for processed material and adulteration or substitution is documented across the commercial supply chain [38,41,42]. Molecular approaches such as DNA barcoding and metabarcoding are informative for raw and minimally processed material but lose sensitivity in highly refined extracts, where chemical fingerprinting becomes the more appropriate tool; the two approaches are therefore complementary rather than interchangeable [39,41]. Studies combining pharmacobotanical description with phytochemical profiling of a defined regional population illustrate the level of characterization the framework requires [43], while micromorphological description of secretory structures provides complementary anatomical criteria applicable to vegetative organs, which retain diagnostic value for raw material but not for refined extracts [44]. Variety and cultivation regime likewise modify the constituent profile of plant-derived oils, and should therefore be recorded alongside species identity whenever the plant material is agriculturally produced [45].

Beyond identity, the extract must be traceable to a defined process. Plant part, geographical origin and harvest period, drying and storage conditions, extraction solvent and its concentration, drug-to-extract ratio and extraction yield all influence the constituent profile and therefore the measured antimicrobial and cytotoxic endpoints [37,46]. A chromatographic fingerprint obtained by high-performance liquid chromatography or liquid chromatography coupled to mass spectrometry, together with quantification of selected marker compounds, provides the analytical identity against which subsequent batches are compared [39,40]. Selection of markers should be justified in relation to the presumed mechanism of activity rather than to analytical convenience, and the criteria proposed for ranking chemical markers in complex herbal materials offer a transparent basis for that choice [40].

### 5.3. Essential Oils: Chemotype, Composition and Oxidative Status

For essential oils, the botanical species alone is insufficient to define the test material, because a single species may comprise several chemotypes whose dominant constituents, and therefore whose antimicrobial potency and cytotoxic profile, differ substantially. The chemotype must be stated explicitly and supported by a quantitative compositional profile obtained by gas chromatography with mass spectrometric and flame ionization detection, with the relative abundance of major constituents reported [17,47].

Oxidative status deserves particular emphasis and is rarely reported. Monoterpenes and other constituents undergo autoxidation on exposure to air, light and elevated temperature, generating hydroperoxides and secondary products that are both less active antimicrobially and more irritant and sensitising than the parent compounds [17]. An essential oil tested after prolonged or uncontrolled storage is not the material described by its original certificate of analysis. Storage conditions, headspace, light exposure and the interval between analysis and testing should therefore be documented, and compositional analysis should be repeated when this interval is prolonged. The container itself is part of these conditions, since the compatibility of an essential-oil-containing preparation with its primary packaging influences stability over time [48]. As a methodological safeguard proposed here on the basis of the literature synthesized, batch reproducibility should be demonstrated on at least two independent lots before formulation development is initiated [17,47]. The composition to which microorganisms are exposed also depends on the phase in which the oil is presented, since the vapour phase is enriched in the more volatile constituents relative to the liquid phase and the two may differ substantially in the abundance of the compounds held responsible for activity; where vapour-phase exposure is intended, the profile of that phase should be characterized rather than inferred from the liquid-phase analysis [49].

### 5.4. Honey: Origin, Grade and Physicochemical Determinants of Activity

Honey requires a distinct characterization set because its antibacterial activity arises from several partly independent mechanisms whose relative contribution varies with origin and processing [33,50]. Botanical and geographical origin should be stated, together with whether the material is of medical grade, since medical-grade products are standardized for antibacterial activity and sterilized by gamma irradiation, whereas retail honeys are not and may differ substantially in performance [50,51]. Comparative analyses of monofloral honeys show that phenolic composition and antioxidant capacity vary with botanical origin and with the acquisition source [52], and that physicochemical parameters differ measurably between supply chains for the same declared floral type [53]; neither variable is inferable from the product label alone.

The physicochemical determinants that should accompany any antimicrobial dataset are moisture content and water activity, pH, and sugar profile, since the osmotic component of activity is a direct function of these parameters. Peroxide-dependent and non-peroxide activity should be distinguished experimentally, typically by comparison of activity in the presence and absence of catalase, and methylglyoxal content should be reported where non-peroxide activity is claimed [33,50]. The sterilization method must be stated, as it may modify both enzymatic and non-enzymatic components. Reporting a single inhibitory concentration for an uncharacterized honey conveys little transferable information, and the same limitation applies to comparisons between honeys characterized to different depths [50].

### 5.5. Sugar and Vehicle Controls as an Extension of Characterization

Pre-analytical characterization and assay control are continuous rather than separate activities. For honey, an artificial honey control matching the sugar composition and concentration of the test material is required to separate the osmotic contribution from specific antibacterial mechanisms. For essential oils, the emulsifier or dispersing agent must be tested at the identical final concentration used in test wells. For extracts, the solvent control must match both composition and proportion. In each case, the control is defined by the characterization data generated at this stage, which is a further reason for placing standardization before, rather than alongside, antimicrobial testing [3,16,50].

## 6. Methods for the Evaluation of Antimicrobial Activity of Natural Products: Experimental Principles, Technical Execution, Controls, Sources of Error and Formulation-Oriented Interpretation

### 6.1. Conceptual Positioning of Antimicrobial Testing in the Development of Topical Natural Products

The evaluation of antimicrobial activity in plant extracts, essential oils, and honey intended for topical use must be approached as a staged experimental process, in which each method contributes a specific layer of information, and the overall relevance emerges only through coherent integration of these data [3,14,16]. In contrast to synthetic antimicrobials, natural products represent complex matrices with variable physicochemical properties that directly influence assay performance, result interpretation, and translational relevance.

From a formulation-oriented perspective, antimicrobial testing does not aim merely to demonstrate activity, but to generate parameters that can inform feasibility, safety margins, and technological decisions. Consequently, no single method is sufficient to support development decisions. Instead, screening, quantitative, and confirmatory approaches must be combined within a controlled methodological framework [2,6,17].

### 6.2. Preliminary Screening of Antimicrobial Activity by Agar Diffusion Methods

Agar diffusion assays represent the first level of antimicrobial evaluation and are used exclusively for preliminary screening of potential activity. The method involves uniform inoculation of a suitable solid culture medium with a standardized bacterial suspension, followed by application of the test product onto sterile discs or into wells cut into the agar. After incubation at 35 ± 1 °C for 18–24 h, inhibition zones are measured as indicators of growth suppression [3,16].

In the context of natural products, interpretation of agar diffusion results is strongly influenced by physicochemical characteristics. Hydroalcoholic plant extracts may diffuse relatively uniformly through the agar matrix, whereas essential oils can generate inhibition zones partly due to volatility rather than true diffusion. Highly viscous matrices such as honey or concentrated extracts may diffuse poorly, resulting in small or absent inhibition zones despite demonstrable antimicrobial activity in liquid systems [3,8,10].

For these reasons, the absence of an inhibition zone must not be interpreted as a lack of biological activity, and the presence of a large zone does not guarantee pharmacological relevance. Agar diffusion assays provide qualitative, indicative information only and are not suitable for quantitative comparison or formulation decision-making. Their value lies in identifying candidates warranting further investigation using standardized quantitative methods [3,16].

### 6.3. Determination of Minimum Inhibitory Concentration by Broth Microdilution

Broth microdilution is the central quantitative method for evaluating antimicrobial activity of natural products. The assay is based on preparing serial dilutions of the test product in an appropriate liquid culture medium, followed by inoculation with a standardized bacterial suspension, typically adjusted to yield a final concentration of approximately 5 × 10^5^ colony-forming units per milliliter. After incubation under controlled conditions, the minimum inhibitory concentration is defined as the lowest concentration at which no visible bacterial growth is observed [1,2,15,18].

For natural products, ensuring adequate solubility and homogeneous dispersion represents a critical methodological step. Plant extracts must be fully dissolved, essential oils require emulsification using agents that are microbiologically inert at the applied concentrations, and honey must be diluted sufficiently to allow uniform mixing without abolishing biologically relevant mechanisms such as osmotic stress [10,15]. Each experiment must include a solvent control identical in composition and proportion to that used in test wells, as omission of this control precludes valid attribution of observed effects to the natural product itself [16,17]. A published step-by-step protocol for the determination of inhibitory concentrations of essential oils by broth microdilution, including dispersion and inoculum standardization, provides a reference implementation of these requirements and illustrates the absence of a harmonized reference assay that the framework seeks to address [54].

Reading of results may be complicated by intrinsic turbidity or coloration of natural matrices. In such cases, visual assessment should be complemented by subculture on solid media or by compatible metabolic indicators to confirm inhibition of bacterial growth. MIC values obtained under these conditions must be interpreted as relative indicators of antimicrobial potency rather than absolute thresholds of efficacy or safety [3,14].

### 6.4. Determination of Minimum Bactericidal Concentration and Interpretation of Inhibitory–Bactericidal Relationships

The minimum bactericidal concentration (MBC) is determined by subculturing samples from wells showing no visible growth in the microdilution assay onto solid agar media, followed by incubation and assessment of bacterial viability. A reduction of at least 99.9% relative to the initial inoculum is generally considered indicative of bactericidal activity [2,18].

In the case of natural products, strict differentiation between bacteriostatic and bactericidal effects requires cautious interpretation. Many natural matrices induce reversible metabolic inhibition or sublethal damage that does not immediately translate into bacterial death. For topical applications, where exposure may be repeated or prolonged, sustained inhibition may be sufficient to control infection even in the absence of rapid bactericidal activity [6].

Accordingly, the relationship between minimum inhibitory and minimum bactericidal concentrations should be interpreted in light of the intended application, exposure duration, and formulation strategy. Overemphasis on bactericidal classification may undervalue products that are nonetheless effective in clinically relevant topical contexts [3].

### 6.5. Time–Kill Assays and Assessment of Antimicrobial Dynamics

Time–kill assays provide essential information on the dynamics of antimicrobial activity by monitoring changes in bacterial population size over time under exposure to defined concentrations of the test product. Bacteria are exposed to concentrations typically corresponding to the MIC or multiples thereof, and viable counts are determined at predefined intervals by enumeration of colony-forming units [34,55].

This approach allows differentiation between products that exert rapid but transient effects and those that maintain sustained inhibition. Regrowth phenomena, frequently observed with natural products, cannot be captured by static endpoint assays and have direct implications for dosing frequency and formulation design [17,34]. Interpretation of time–kill data must consider the stability of the product in the test system and the potential influence of solvent or dispersing agents over time.

### 6.6. Evaluation of Antibiofilm Activity and Relevance for Topical Applications

Biofilms represent a highly relevant mode of microbial growth in chronic wounds and cutaneous infections, characterized by increased tolerance to antimicrobial agents. Evaluation of antibiofilm activity is therefore critical for assessing the translational value of natural products intended for topical use [34,35,55,56].

In vitro biofilm models quantify biomass or metabolic activity using colorimetric or metabolic assays. It is essential to distinguish between inhibition of biofilm formation and disruption of established biofilms, as these represent distinct biological phenomena. Many natural products are effective in preventing initial adhesion but show limited activity against mature biofilm structures [34,35].

While in vitro biofilm models necessarily simplify biological reality, they provide valuable comparative information when interpreted cautiously and integrated with other antimicrobial and cytotoxicity data. Their inclusion enhances the relevance of antimicrobial evaluation for real-world topical applications.

### 6.7. Antifungal Susceptibility Testing of Natural Products

Topical infections of the skin and mucosa are frequently fungal, and a framework that claims antimicrobial rather than antibacterial scope must address fungal endpoints explicitly. Yeasts, dermatophytes and other filamentous fungi differ sufficiently in growth kinetics and morphology that a single protocol cannot serve all three, and dedicated reference methods exist for broth dilution testing of yeasts and of filamentous fungi [21,22]. These reference methods define the growth medium, inoculum preparation and density, incubation temperature and duration, and the endpoint criteria applicable to each group, and they should be the starting point for testing of natural products in the same way that bacterial reference methods are.

Three points require specific attention when natural matrices are tested. First, endpoint definition differs from the bacterial setting: partial inhibition of growth is common with azoles and with several natural products, so the proportion of growth reduction used to define the endpoint must be stated rather than assumed, and visual reading should be supported by a spectrophotometric or metabolic readout where matrix colour or turbidity interferes. Second, dermatophytes require prolonged incubation, during which volatile constituents of essential oils may be substantially depleted; closed systems or sealed plates are therefore necessary to avoid underestimating activity, while the same volatility may cause overestimation in adjacent wells if plates are not sealed appropriately [57]. Third, morphological transition and biofilm formation by *Candida* species are biologically distinct endpoints from planktonic inhibition and are frequently the more relevant ones for mucosal and cutaneous indications; several natural products inhibit filamentation and biofilm development at concentrations below those required for planktonic inhibition [58].

A trade-off arises where sealed systems are used with organisms requiring prolonged incubation. Dermatophytes and other filamentous fungi are obligate aerobes, and a seal tight enough to retain volatile constituents over several days may restrict gas exchange sufficiently to suppress growth independently of the test product, producing an apparent inhibition indistinguishable from genuine antifungal activity. The framework therefore requires that a sealed growth control containing no test product be included on the same plate and under the same seal, since suppression of growth in that control is attributable to the test system rather than to the product; that growth in the sealed control be compared with an unsealed control incubated in parallel wherever feasible, and the inhibitory concentration interpreted only where the two are comparable; that seal type, plate format and headspace volume be reported as part of the method, since they determine the magnitude of the effect; and that viability at the endpoint be confirmed by subculture rather than inferred from the absence of visible growth. Where the intended readout is vapour-phase activity, assay formats developed specifically for that purpose, including sealing devices validated against established volatilization assays, are preferable to improvised sealing [59].

Where the intended indication is a specific mycosis, the reference strain panel should include species that are epidemiologically relevant to that indication, and susceptibility should be interpreted against the same quality-control logic applied to bacterial testing rather than against clinical breakpoints, which are not transferable to natural products [21,22,57].

### 6.8. Biofilm Endpoints: From Qualitative Antibiofilm Activity to Defined Parameters

The term antibiofilm activity is used in the literature for biologically distinct phenomena that carry different translational implications, and the resulting data are frequently not comparable [60,61,62]. The framework therefore requires that the phenomenon under study be specified as one of the following: inhibition of initial adhesion, inhibition of biofilm formation when the product is present during development, or activity against an established biofilm of stated maturity. The age of the biofilm at the time of exposure should always be reported, since tolerance increases markedly with maturation and results obtained on biofilms of a few hours cannot be extrapolated to the chronic wound setting [61,63].

Endpoint parameters should be named according to what they measure. The minimum biofilm inhibitory concentration expresses the lowest concentration preventing biofilm formation, whereas the minimum biofilm eradication concentration expresses the lowest concentration eliminating viable cells from an established biofilm; the two are not interchangeable and typically differ by one or more orders of magnitude [61,62]. Reporting both, where the method permits, conveys considerably more information than a percentage reduction at a single concentration.

The choice of readout requires explicit justification. Crystal violet staining quantifies total biomass, including matrix and non-viable cells, and therefore cannot by itself demonstrate killing; metabolic assays report activity rather than biomass, and may underestimate persister populations; enumeration of colony-forming units after disaggregation reports viable cells but is sensitive to the disaggregation procedure. Because each readout answers a different question, the framework proposes that at least two complementary readouts be used before an antibiofilm claim is made, and this requirement is carried into the minimum evidence for Gate 3 in Section 9.8 [61]. Carry-over of the test product into the recovery medium is a specific and frequently overlooked source of error in biofilm and time-kill work: if the product continues to act after transfer, killing is overestimated, and a validated neutralization or recovery procedure is therefore required.

Finally, chronic wound infections are typically polymicrobial, and interspecies interactions modify both tolerance and virulence in ways that monospecies models cannot capture [63,64]. The clinical counterpart of this complexity is that infection in a chronic ulcer is frequently difficult to establish on clinical grounds alone, and inflammatory markers have been examined as adjuncts to diagnosis in infected diabetic foot ulcers [65]; the organisms recovered in such settings are the ones against which a topical adjuvant would ultimately have to act. Where the intended indication is the chronic wound, evaluation in a polymicrobial model, for example combining a Gram-positive and a Gram-negative species relevant to that setting, materially strengthens translational relevance, and its absence should be acknowledged as a limitation rather than left unstated [64].

### 6.9. Integration of Antimicrobial Methods and Control of Experimental Error

The true value of antimicrobial evaluation emerges only through coherent integration of complementary methods. Preliminary screening identifies promising candidates, quantitative assays provide standardized potency parameters, bactericidal and kinetic assays clarify the nature and stability of the effect, and antibiofilm models add clinically relevant context [3,16,17,34,35,55,56].

Common sources of experimental error include absence of solvent controls, misinterpretation of intrinsic turbidity as growth inhibition, volatility-related overestimation of essential oil activity, and unjustified extrapolation of in vitro results to clinical efficacy. Rigorous control of these factors is indispensable if antimicrobial data are to inform formulation decisions responsibly [16,17].

The principal methods, the information each provides and their main limitations are summarized in Table 2. The most frequent sources of error and the corresponding control measures are listed in Table 3, while the interferences specific to each matrix class, together with the required controls and the recommended solutions, are set out in Table 4. The minimum information that should accompany any report of antimicrobial testing of a natural product intended for topical use is proposed in Table 5.

## 7. Quality Control in Antimicrobial Testing of Natural Products: Reference Strains, Method Validation and Data Traceability

### 7.1. The Central Role of Quality Control in Natural Product Antimicrobial Testing

Quality control represents a fundamental component of antimicrobial evaluation and acquires heightened importance in the context of natural products, where compositional variability and matrix complexity can profoundly influence experimental outcomes. Unlike synthetic antimicrobials, which can often be treated as chemically homogeneous systems, plant extracts, essential oils, and honey are intrinsically heterogeneous and subject to variation arising from biological origin, extraction procedures, and storage conditions [3,4,30]. Without a rigorous quality control framework, experimental results may reflect methodological artefacts rather than true biological activity.

Quality control in this context should not be understood as a formal or peripheral requirement, but as an active process of method validation and risk mitigation. It encompasses verification of inoculum standardization, performance of culture media, stability of test conditions, appropriateness of solvent controls, and the consistent behavior of reference strains across experiments [1,2,18]. Together, these elements ensure that observed antimicrobial effects can be attributed with confidence to the tested product.

### 7.2. Reference Strains as Anchors of Methodological Validity

Reference strains obtained from internationally recognized culture collections constitute the cornerstone of quality control in antimicrobial susceptibility testing. These strains are genetically and phenotypically stable and are characterized by well-documented susceptibility profiles, allowing them to serve as internal benchmarks for method performance [1,2,18].

In the evaluation of natural products, reference strains are not employed to define clinical susceptibility breakpoints, but to confirm that the experimental system is capable of detecting antimicrobial activity when present and reproducing consistent results across independent assays. Their inclusion allows discrimination between true absence of activity and methodological failure, particularly in cases where natural matrices interfere with assay readouts [3,16].

### 7.3. Selection of Reference Strains and Biological Relevance for Topical Use

The selection of reference strains must be aligned with the intended application of the natural product. For topical formulations, inclusion of microorganisms commonly implicated in cutaneous and mucosal infections is essential, as is representation of both Gram-positive and Gram-negative bacteria with distinct cell envelope structures [1,6]. Differences in membrane composition and intrinsic resistance mechanisms can markedly influence susceptibility to natural antimicrobials, particularly those acting at the membrane level [8,9].

Incorporation of strains known for intrinsic tolerance or biofilm-forming capacity further enhances the relevance of quality control, as these organisms can expose methodological limitations and provide insight into the potential translational performance of the product [34,35,55,56]. Selection criteria should be explicitly documented and justified in relation to the target indication and existing literature [4,5].

### 7.4. Control of Bacterial Inoculum and Incubation Conditions

Inoculum density is a critical determinant of antimicrobial assay outcomes. Excessively high inocula may obscure antimicrobial effects, whereas overly dilute suspensions can exaggerate apparent activity. Standardization of inoculum density is therefore mandatory and must be verified periodically using quantitative methods to ensure consistency across experiments [1,2,18].

Incubation conditions, including temperature, duration, and atmospheric composition, must be strictly controlled and documented. Even minor deviations can alter bacterial growth kinetics and introduce artificial variability into results. For natural products, where effects may be subtle or concentration-dependent, such variability can have disproportionate impact on interpretation [3,15]. Continuous monitoring and documentation of incubation parameters are essential components of data traceability.

### 7.5. Solvent and Vehicle Controls as Critical Safeguards

Solvents and vehicles represent one of the most significant sources of potential bias in natural product testing. Many solvents required for solubilization or dispersion of natural matrices possess intrinsic antimicrobial or cytotoxic properties, which can confound attribution of observed effects if not properly controlled [16,17].

Implementation of solvent controls identical in composition and proportion to those used in test wells is mandatory for every experiment. Interpretation of antimicrobial activity must be performed exclusively by comparison with these controls. Absence or inappropriate design of solvent controls is a common methodological flaw and a major contributor to false-positive findings in the literature on natural antimicrobials [3,16].

### 7.6. Reproducibility, Repeatability and Internal Validation

An effective quality control system requires assessment of both repeatability and reproducibility. Repeatability is evaluated through technical replicates within the same experiment, while reproducibility is assessed by repeating assays across independent experiments performed on different days or with different product batches [1,2,18].

Consistent results across replicates indicate robustness of the method and reliability of the data generated. In contrast, high variability may signal instability of the natural product, inadequate standardization of experimental parameters, or insufficient control of matrix-related interferences. Identification and correction of such issues are integral to internal validation and essential for supporting downstream formulation decisions [4,30].

### 7.7. Tiered Strain Selection: From Method Validation to Translational Relevance

Reference strains establish that the experimental system performs as intended, but they cannot by themselves establish that a product will be active against the organisms encountered in practice. Collection strains have been maintained under laboratory conditions for extended periods and differ from contemporary clinical isolates in virulence determinants, biofilm-forming capacity and resistance profile. A framework whose stated justification is antimicrobial resistance cannot rest on reference strains alone [61,63].

The framework therefore proposes a tiered strain panel. The first tier comprises reference strains from internationally recognized collections and serves method validation and quality control; results at this tier confirm that the system detects activity when present and reproduces it across independent runs [1,2,18]. The second tier comprises contemporary clinical isolates relevant to the intended indication and serves translational confirmation; activity at this tier indicates that the effect observed under controlled conditions is retained against organisms currently circulating in the target setting. The third tier comprises isolates with documented resistance phenotypes and serves to establish relevance to the antimicrobial resistance problem that motivates development.

The tiers are sequential rather than alternative. Testing clinical isolates without a validated reference-strain baseline produces data that cannot be interpreted when activity is absent, since methodological failure and true inactivity become indistinguishable. Conversely, activity confirmed only against reference strains should be reported as such, and claims of relevance to resistant infection should not be made in its absence. The provenance, year of isolation and resistance profile of clinical isolates should be documented, together with the ethical approval covering their use where applicable.

### 7.8. Traceability and Documentation of Experimental Data

Traceability is ensured through comprehensive documentation of all experimental parameters, including source and batch of the natural product, extraction method, solvent composition, culture media, inoculum preparation, incubation conditions, and data analysis procedures. This documentation enables critical evaluation of results, supports reproducibility, and facilitates meaningful comparison across studies [20].

For development-oriented research, traceability also underpins regulatory compliance and risk assessment. Data lacking clear provenance or methodological transparency are of limited value for translational decision-making, regardless of apparent antimicrobial potency [6].

## 8. Integration of Minimum Inhibitory Concentration, Minimum Bactericidal Concentration and Cellular Cytotoxicity: Rationale and Application of the Selectivity Index as a Decision-Making Tool

### 8.1. Limitations of Antimicrobial Potency as a Standalone Criterion

Evaluation of natural products with antimicrobial potential intended for topical use cannot be considered complete when based solely on antimicrobial potency parameters such as minimum inhibitory concentration or minimum bactericidal concentration. While these parameters provide essential information regarding the ability of a product to inhibit or eliminate microorganisms under standardized experimental conditions, they do not offer insight into the biological cost of achieving such effects at the level of host tissues [2,3,14]. In topical applications, where local concentrations may be substantially higher than those encountered in systemic therapy, neglecting cellular safety can lead to formulations that are microbiologically effective but clinically unacceptable.

The disconnect between antimicrobial activity and host–cell tolerability represents a critical translational barrier frequently encountered in the literature on natural antimicrobials. Products are often advanced on the basis of low MIC values without consideration of whether these concentrations can be safely applied to skin or mucosal surfaces [6,17]. This limitation underscores the necessity of integrating antimicrobial and cytotoxicity data into a unified evaluative framework.

### 8.2. Cellular Models Relevant to Topical Applications

Assessment of cytotoxicity must be performed using cellular models that are biologically relevant to the intended application. For topical formulations, keratinocytes and dermal fibroblasts are commonly employed, as they represent the primary cell types exposed during cutaneous application. For mucosal applications, epithelial cell lines derived from oral, vaginal, or intestinal tissue may be more appropriate, depending on the target indication [17].

Cytotoxicity is typically quantified using viability assays that measure metabolic activity, membrane integrity, or cellular proliferation, allowing determination of the concentration at which cell viability is reduced by fifty percent, commonly referred to as the half-maximal cytotoxic concentration (CC50). This parameter provides a quantitative estimate of cellular tolerance under controlled experimental conditions and serves as a critical counterbalance to antimicrobial potency metrics [17].

### 8.3. Integrating Cytotoxicity Data with Antimicrobial Endpoints

Reporting cytotoxicity data in isolation is insufficient to guide development decisions. A natural product may exhibit minimal cytotoxicity at low concentrations yet lack antimicrobial efficacy, or conversely, demonstrate potent antimicrobial activity only at concentrations associated with unacceptable cellular toxicity. To resolve this ambiguity, antimicrobial and cytotoxicity endpoints must be evaluated in relation to one another rather than independently [2,17].

This integration requires that cytotoxicity assays be conducted under conditions comparable to antimicrobial testing, including use of identical solvents or vehicles and comparable exposure durations. Solvent effects must be carefully controlled, as solvents may contribute independently to cytotoxicity and distort interpretation if not properly accounted for [16,17]. Only through such methodological alignment can meaningful integration of datasets be achieved.

### 8.4. Definition and Rationale of the Selectivity Index

The Selectivity Index is defined as the ratio between the concentration inducing a fifty percent reduction in host–cell viability (CC50) and the minimum inhibitory concentration against the target microorganism. This ratio provides a quantitative estimate of the safety margin separating antimicrobial efficacy from cellular toxicity [2,17].

A high Selectivity Index indicates that antimicrobial activity is achieved at concentrations substantially lower than those associated with host–cell damage, suggesting favorable translational potential for topical use. Conversely, a low Selectivity Index reflects a narrow or absent safety margin, indicating that antimicrobial efficacy is closely coupled to cytotoxicity and that direct application in its current form may be unsafe.

The conceptual value of the Selectivity Index lies in its integrative nature. Rather than treating efficacy and safety as separate dimensions, it combines them into a single parameter that can inform rational decision-making early in the development process.

### 8.5. Interpretation of Selectivity Index Values in a Formulation-Oriented Context

Interpretation of Selectivity Index values must be context-dependent and aligned with the intended mode of use. For topical products applied to intact skin for short durations, moderate Selectivity Index values may be acceptable, particularly if formulation strategies can mitigate local exposure or control release kinetics. In contrast, applications involving compromised skin barriers, mucosal surfaces, or repeated long-term use require more conservative thresholds and higher Selectivity Index values to ensure tolerability [6].

Intermediate Selectivity Index values should not be interpreted as automatic exclusion criteria. Instead, they may signal the need for formulation-driven optimization, such as encapsulation, controlled release, or use of protective vehicles that reduce direct cellular exposure while preserving antimicrobial efficacy [6,17]. In this way, the Selectivity Index functions not only as a screening parameter but also as a guide for formulation strategy.

To assist laboratory standardization, an orientation scale drawn from the recent literature on essential oil selectivity may be applied. That literature distinguishes four bands: a Selectivity Index of at least 100, corresponding to a wide margin between inhibitory and cytotoxic concentrations; a value between 10 and 99, regarded as relatively safe but requiring closer control of the applied concentration; a value between 1 and 9, corresponding to a limited margin in which small increases in concentration or exposure duration may produce host–cell injury; and a value below 1, in which the cytotoxic concentration lies at or below the inhibitory concentration [66,67]. Mapped onto the stage-gate criteria set out in Section 9.8, the two upper bands support a Go outcome at Gate 5 where the remaining requirements of that gate are met, the band between 1 and 9 corresponds to a Conditional Go in which advancement depends on a formulation strategy capable of widening the margin, and a value below 1 is a No-Go. This scale is descriptive of prevailing practice rather than a safety criterion. It does not supersede the requirement stated in Section 8.7 that acceptability be defined in advance for each indication, and it should be applied with the caution that indices derived from cancer-derived cell lines may overstate toxicity relative to clinical experience [66].

### 8.6. Methodological Considerations and Sources of Bias

Accurate determination of the Selectivity Index depends on rigorous control of methodological variables. Differences in exposure time, assay sensitivity, or solvent composition between antimicrobial and cytotoxicity assays can introduce bias and artificially inflate or deflate the calculated ratio [16,17]. Consistency in experimental design is therefore essential.

Additionally, intrinsic properties of natural products, such as color or viscosity, may interfere with cytotoxicity assay readouts in a manner analogous to their effects on antimicrobial assays. These interferences must be identified and addressed through appropriate controls and complementary readouts to avoid misinterpretation.

### 8.7. Methodological Requirements for Reporting the Selectivity Index

Because the Selectivity Index occupies a central position in this framework, its calculation and reporting require the same rigour as the underlying assays. Recent syntheses applying the index to essential oils tested against clinically relevant pathogens illustrate both its discriminating value and the heterogeneity of current reporting practice [66,67]. The following requirements are proposed as a minimum.

The index should be expressed as the ratio of the half-maximal cytotoxic concentration to the minimum inhibitory concentration, using the abbreviation CC50 rather than terminology borrowed from enzyme inhibition, which is a distinct concept. Both terms of the ratio must be expressed in identical units, and for multicomponent matrices the unit should refer to the whole extract or oil rather than to a marker constituent unless the tested material is that constituent in isolation. Ratios calculated from values expressed in different units, or from an inhibitory concentration determined in one laboratory and a cytotoxic concentration taken from another study, are not interpretable [68].

The index should be reported separately for each microorganism tested rather than as a single value derived from a pooled or lowest inhibitory concentration, since selectivity is organism-dependent and pooling conceals the variation that matters for indication-specific development. Where bactericidal activity is relevant to the intended use, a corresponding ratio based on the minimum bactericidal concentration may be reported alongside, and labelled explicitly as such. The cell line and exposure duration used to derive CC50 must be stated, together with the assay endpoint, and exposure conditions should match those of the antimicrobial assay as closely as the biology permits; a ratio derived from a 24-h antimicrobial endpoint and a 72-h cytotoxicity endpoint quantifies partly the difference in exposure time [68].

CC50 values should be reported with 95% confidence intervals derived from the fitted concentration-response curve, and the number of independent biological replicates should be stated. A ratio calculated from point estimates without dispersion cannot support comparison between candidates, since apparent differences may lie entirely within the uncertainty of the underlying fits.

Finally, no universally valid threshold separates acceptable from unacceptable selectivity across products and indications, and the framework deliberately refrains from proposing one. Descriptors such as favourable, intermediate and unfavourable are interpretive rather than absolute and must be defined in relation to the intended site of application, the integrity of the barrier at that site, the duration and frequency of exposure and the population concerned. A value acceptable for short-term application to intact skin may be unacceptable for repeated application to an ulcerated surface. Presenting the index as a numerical threshold above which a product is safe misrepresents both its derivation and its predictive value [66,67].

### 8.8. From Cellular Cytotoxicity to Topical Tissue Tolerability

Viability assays on submerged monolayers of keratinocytes or dermal fibroblasts quantify a necessary but not a sufficient component of topical safety. Such models lack a stratum corneum, do not reproduce barrier function, and expose cells to the test material at a concentration and in a manner that bear little relationship to application onto skin. Products that appear cytotoxic in monolayer may be well tolerated when applied topically, and the converse also occurs [69]. Cellular cytotoxicity is therefore the entry point to safety assessment rather than its conclusion.

A graded progression is proposed. Monolayer assays on keratinocytes and fibroblasts serve concentration ranging and the derivation of CC50 for the Selectivity Index. Reconstructed human epidermis models, which reproduce a stratified and cornified epithelium, then permit assessment of irritation potential under topically relevant exposure using an internationally accepted test guideline, and are the appropriate level at which to evaluate a prototype formulation rather than a raw material. It should be emphasized that this guideline identifies irritation hazard and does not by itself establish clinical tolerability in a given indication, for which it serves as a safety screening stage [23,69,70]. Where the intended indication involves a compromised barrier, models incorporating barrier disruption are more informative than intact models, since pre-existing inflammatory skin disease and cutaneous trauma may both predispose to severe soft-tissue infection [71], and barrier integrity endpoints such as transepithelial electrical resistance add interpretable information about whether the product itself impairs the barrier it is intended to protect.

Sensitisation is a separate endpoint from irritation and is not predicted by cytotoxicity. It is particularly pertinent to essential oils, whose constituents include recognized contact allergens and whose oxidation products are frequently more sensitising than the parent molecules, which links this assessment directly to the oxidative status documented at the pre-analytical stage [17,72]. Non-animal approaches addressing the key events of the sensitisation pathway are available and have been applied specifically to botanical ingredients, and defined approaches combining in chemico and in vitro endpoints are now formalized in an international guideline [26,70,72]. For products intended for photoexposed skin, and for matrices containing furocoumarins or other photoactive constituents, phototoxicity assessment should be added. Reporting a natural product as safe on the basis of a single viability assay is not defensible for a topical indication, and the framework treats irritation and sensitisation endpoints as decision-relevant rather than optional.

### 8.9. Pathogen Selectivity Relative to the Resident Skin Microbiota

A framework built on the argument that indiscriminate antimicrobial exposure is harmful should apply the same reasoning to the site of application. The resident cutaneous microbiota is not a passive bystander: commensal staphylococci contribute directly to barrier homeostasis, including through lipid metabolism, and to colonization resistance against pathogens [73,74]. Systemic antibiotic exposure is now recognized to cause collateral damage to commensal communities with downstream clinical consequences, and there is no reason to assume that broad-spectrum topical antimicrobials are exempt from an analogous effect at the cutaneous surface [75].

The framework therefore proposes that evaluation address not only whether a product inhibits the target pathogen, but whether it does so preferentially relative to relevant commensals. In practice this means including at least one commensal species appropriate to the indication in the test panel alongside the target pathogen, and reporting the inhibitory concentrations side by side. The choice is indication-dependent: in acne, for example, the target organism is also a resident of healthy skin, so indiscriminate suppression is not a therapeutic goal [74]. Interest in non-conventional therapeutic approaches for this indication is correspondingly substantial [76]. A product that inhibits the pathogen at concentrations substantially below those affecting the commensal offers a different translational proposition from one that suppresses both equally, particularly where use is prolonged or repeated.

No validated numerical index of microbiome selectivity currently exists, and the framework deliberately does not propose one, since a ratio derived from two planktonic inhibitory concentrations would substantially oversimplify a community-level phenomenon that also involves interspecies interaction and host response [63,74]. What is proposed is a decision criterion and a reporting requirement: the comparative data should be generated and presented, and the implications for the intended duration of use should be discussed explicitly. This is an area in which the framework identifies a gap rather than closing it, and in which method development is needed.

As an exploratory reporting convention rather than a validated parameter, the comparison may be expressed as a commensal sparing ratio, defined as the inhibitory concentration against the commensal species divided by the inhibitory concentration against the target pathogen, both determined in the same experiment under identical conditions and reported for each pathogen-commensal pair. A value above unity indicates that the pathogen is inhibited at lower concentrations than the commensal; a value near unity indicates indiscriminate inhibition; a value below unity indicates that the commensal is the more susceptible of the two, which is the least favourable case where use is prolonged. Three constraints are inseparable from this proposal. No cut-off value is proposed, since none is supported by current evidence. The ratio is derived from planktonic monoculture data and cannot represent a community-level phenomenon that also involves interspecies interaction and host response. And it is interpretable only where both terms are generated under matched conditions, for the same reasons that apply to the Selectivity Index in Section 8.7. So constrained, the ratio makes the gap measurable and comparable between studies without implying that it has been closed.

## 9. Go, Conditional Go and No-Go Decision Criteria for Advancing Natural Antimicrobial Products Toward Topical Pharmaceutical Formulation

### 9.1. From Biological Evaluation to Development Decisions

The transition from laboratory evaluation of natural antimicrobial products to pharmaceutical formulation represents a critical inflection point in the development pathway. At this stage, decisions must be grounded in integrated interpretation of antimicrobial efficacy, cellular safety, methodological robustness, and technological feasibility, rather than in isolated experimental outcomes [2,6]. Without explicit decision criteria, there is a substantial risk of advancing candidates that appear promising in vitro but fail during formulation, stability testing, or early translational stages.

Go, Conditional Go and No-Go criteria provide a structured framework that transforms heterogeneous experimental data into actionable development decisions. In the context of topical natural products, these criteria must reflect not only antimicrobial potency but also selectivity, reproducibility, and compatibility with formulation strategies [6,17].

### 9.2. Core Criteria Supporting a Go Decision

A Go decision is supported when antimicrobial activity is demonstrated consistently and reproducibly using standardized quantitative methods. Minimum inhibitory concentration values must fall within concentration ranges that are realistically achievable in topical formulations without compromising tolerability, and results should be confirmed across independent experiments and relevant reference strains [1,2,3,18]. Confirmation through complementary methods, such as time–kill assays or antibiofilm models, strengthens confidence in the robustness and relevance of the observed activity [34,35,55,56].

Equally important is the Selectivity Index, which integrates antimicrobial potency with cellular safety. A favorable Selectivity Index indicates the existence of an adequate safety margin between effective antimicrobial concentrations and those associated with host–cell toxicity, supporting advancement toward formulation [2,17]. Intermediate Selectivity Index values may still justify a Go decision when coupled with feasible formulation strategies capable of modulating local exposure or release kinetics.

### 9.3. Role of Reproducibility and Quality Control in Go Decisions

Reproducibility across experiments and batches represents a non-negotiable criterion for advancement. Natural products exhibiting high variability in antimicrobial activity, cytotoxicity, or physicochemical behavior pose substantial risks during formulation and scale-up [4,30]. Demonstrated consistency under controlled quality assurance conditions supports confidence that observed effects are intrinsic to the product rather than artefacts of experimental variability.

Quality control measures, including performance of reference strains, standardized inoculum preparation, and solvent controls, must confirm that the methodological framework is functioning as intended [1,2,16,18]. A Go decision in the absence of such validation undermines the reliability of downstream development efforts.

### 9.4. Formulation Feasibility as an Integral Decision Parameter

Antimicrobial efficacy and selectivity alone are insufficient to justify advancement if formulation feasibility is questionable. Natural products with favorable biological profiles but poor solubility, instability, or incompatibility with common excipients may still be advanced only if viable technological solutions exist to overcome these limitations [6].

Compatibility with intended dosage forms, stability under anticipated storage conditions, and maintenance of antimicrobial activity within prototype formulations must be considered at this stage. Products that lose activity upon incorporation into simple topical vehicles or that require impractically complex formulation strategies may warrant cautious progression or reconsideration.

### 9.5. Criteria Triggering a No-Go Decision

A No-Go decision is warranted when integrated evaluation reveals an unfavorable balance between antimicrobial efficacy and cellular safety. Products requiring concentrations near or exceeding cytotoxic thresholds to achieve microbial inhibition present unacceptable risks for topical use in their current form [2,17]. Similarly, products whose antimicrobial activity is highly dependent on solvent effects or experimental artefacts should be excluded until these issues are resolved.

Lack of reproducibility, excessive batch-to-batch variability, or inability to standardize composition and performance constitute additional grounds for a No-Go decision. In such cases, further development without addressing these fundamental issues is unlikely to succeed and may divert resources from more viable candidates [4,30].

### 9.6. Context-Dependent and Dynamic Nature of Stage-Gate Decisions

Go, Conditional Go and No-Go criteria should not be interpreted as rigid thresholds but as context-dependent decision tools. Intended clinical application, duration of use, condition of the target tissue, and patient population all influence acceptable risk–benefit profiles. For example, short-term use on intact skin may tolerate narrower safety margins than long-term application on compromised or inflamed tissue [6].

Accordingly, decisions must integrate experimental data with anticipated clinical context and technological constraints. Reassessment may be appropriate if new formulation strategies or additional safety data alter the balance between efficacy and risk.

### 9.7. Conditional Go Decisions and Iterative Development

In many cases, advancement toward formulation occurs under conditional Go decisions, where progression is contingent upon addressing identified limitations. Such conditions may include optimization of extraction procedures, refinement of formulation approaches, or additional cytotoxicity and stability testing [6,17].

This iterative approach acknowledges the developmental nature of early-stage research and leverages formulation science as an active tool for enhancing selectivity and usability, rather than as a passive downstream step.

### 9.8. Formalizing the Conditional Go Decision

Binary Go and No-Go criteria do not reflect the reality of early-stage development of natural products, where candidates frequently combine convincing antimicrobial activity with one identifiable and potentially remediable limitation. The framework therefore formalizes a third outcome, Conditional Go, as an explicit category within the decision structure rather than as an informal practice.

A Go decision requires reproducible inhibitory concentrations across independent experiments and batches, a selectivity margin adequate for the intended site and duration of application, confirmation of activity in a model relevant to the indication, acceptable tolerability in a topically relevant safety model, and a formulation route that is technologically feasible at the anticipated scale.

A Conditional Go decision applies where antimicrobial activity is established and reproducible but progression depends on resolving a defined limitation. Typical situations include an intermediate selectivity margin that may be widened by controlled release or encapsulation, limited aqueous solubility or volatility requiring a specific delivery approach, or oxidative instability requiring reformulation or protective packaging. The condition must be stated as a testable objective with a predefined endpoint, and the candidate is reassessed against the full criteria once the condition has been addressed rather than progressing by default.

A No-Go decision applies where antimicrobial efficacy is achieved only at concentrations approaching or exceeding cytotoxic thresholds, where activity is attributable to the solvent or vehicle rather than to the product, where variability between batches cannot be controlled by feasible standardization, where activity is lost on incorporation into a representative prototype, or where irritation or sensitisation findings are incompatible with the intended use. In these cases further investment is unlikely to be productive unless the underlying issue is addressed at the level of the raw material. The evidence required at each gate, together with the corresponding Go, Conditional Go and No-Go outcomes, is summarized in Table 6.

### 9.9. Worked Application of the Framework to a Single Agent

The framework is presented above as a sequence of criteria. To make the decision logic visible in practice, this subsection traces a single well-documented agent, medical-grade manuka honey, through the seven gates. The agent was chosen because its evidence base is unusually complete across all seven domains, which allows the reasoning to be followed without the discussion becoming an argument about the underlying data. The exercise is a retrospective illustration assembled from published evidence and not a prospective validation of the framework; the limitation stated in Section 11.3 is unaffected by it. For the same reason, Table 7 reports the kind of evidence available at each gate and the decision it supports, rather than individual inhibitory or cytotoxic values, which vary with the honey and with the assay.

The instructive feature of the example is that a well-documented agent nevertheless does not close every gate. Gates 1, 2, 6 and 7 close with a Go outcome on the published evidence. Gate 3 closes conditionally where a study reports biomass staining alone, since two complementary readouts are required. Gate 5 yields a Conditional Go, because comparative inhibitory data for resident cutaneous commensals are sparse even for this agent. That gap is the one identified in Section 8.9, and its appearance here indicates that it is a general limitation of the field rather than an artefact of less well-characterized candidates.

## 10. Pharmaceutical Formulation Strategies for Topical Natural Antimicrobial Adjuvants: Linking Biological Properties to Technological Feasibility

### 10.1. Formulation as a Translational Interface Between Biology and Technology

The development of a topical pharmaceutical formulation incorporating natural antimicrobial products represents the critical interface between biological efficacy and technological feasibility. At this stage, microbiological potency and cellular safety data must be translated into a controlled, reproducible dosage form capable of delivering the active constituents at effective concentrations while maintaining tolerability and stability [6]. Formulation science thus functions not merely as a technical exercise, but as an integrative discipline that actively shapes the translational success of natural antimicrobial adjuvants. Beyond biological efficacy and technological feasibility, translational success also depends on end-user acceptability. Evidence from preventive health interventions in other therapeutic contexts indicates that perceived acceptability is closely associated with willingness to adopt an intervention, and that practical barriers may limit uptake even when the intervention is otherwise well received [83]. Although not incorporated as a formal gate in this laboratory-oriented framework, end-user acceptability remains a relevant consideration during later-stage formulation and clinical translation.

Natural products introduce specific formulation challenges arising from their complex composition, physicochemical heterogeneity, and susceptibility to degradation. Addressing these challenges requires alignment of formulation strategy with the biological profile of the product, particularly its antimicrobial potency, Selectivity Index, and intended site of application [2,17].

### 10.2. Physicochemical Constraints Imposed by Plant Extracts, Essential Oils and Honey

Plant extracts often contain both hydrophilic and lipophilic fractions with distinct solubility profiles. Preservation of antimicrobial activity requires selection of vehicles that solubilize or disperse active constituents without inducing chemical degradation or precipitation. Inadequate solubilization may lead to uneven distribution of active compounds within the formulation and unpredictable local exposure [4,30].

Essential oils present additional constraints due to volatility, lipophilicity, and sensitivity to oxidation. Uncontrolled evaporation or chemical transformation can result in loss of antimicrobial potency and increased risk of local irritation [8,9,31]. Honey, while comparatively stable, introduces formulation challenges related to viscosity, crystallization, and compatibility with other excipients, all of which can influence release kinetics and patient acceptability [10,33].

### 10.3. Selection of Dosage Form Based on Biological Evaluation

Selection of the appropriate dosage form must be guided by integrated biological data. Natural products exhibiting favorable Selectivity Index values may be incorporated into relatively simple vehicles, such as ointments or gels, which allow achievement of effective local concentrations without compromising cellular tolerability [6]. Conversely, products with intermediate Selectivity Index values require formulation strategies that reduce direct cellular exposure while maintaining antimicrobial efficacy.

Bioadhesive vehicles can prolong residence time at the application site, enabling lower concentrations to achieve sustained antimicrobial effects. Controlled-release systems can attenuate peak local concentrations associated with cytotoxicity while maintaining inhibitory levels over extended periods [17]. Thus, dosage form selection is intrinsically linked to the biological safety margin defined during earlier evaluation stages.

### 10.4. Advanced Formulation Approaches: Encapsulation and Nanostructured Systems

Microencapsulation and nanoformulation represent advanced strategies for overcoming biological and physicochemical limitations of natural antimicrobial products. Encapsulation of active constituents within polymeric or lipid-based carriers can shield host cells from direct exposure, enhance chemical stability, and modulate release profiles [6,17].

Such approaches are particularly relevant for essential oils, where volatility and membrane-disruptive properties may otherwise limit safe use. By embedding active components within nanostructured carriers, it is possible to preserve antimicrobial activity while reducing cytotoxicity and irritation potential [17]. However, the complexity of these systems necessitates careful evaluation of carrier biocompatibility, release kinetics, and scalability.

### 10.5. Excipient Compatibility and Preservation of Antimicrobial Activity

Excipients are not biologically inert components; their interaction with natural products can significantly influence antimicrobial efficacy. Certain gelling agents, emulsifiers, or preservatives may bind or inactivate active constituents, alter partitioning behavior, or interfere with microbial assays [6]. Consequently, antimicrobial activity must be reassessed in the context of the final formulation or representative prototypes rather than assumed based on activity of the raw material.

This reassessment serves as a critical validation step, confirming that formulation processes do not negate the biological properties identified during earlier testing. Failure to perform this step represents a common cause of translational failure in natural product development.

### 10.6. Stability Considerations and Their Biological Implications

Chemical and physical stability are essential prerequisites for pharmaceutical viability. Natural products may undergo oxidative, hydrolytic, or photochemical degradation, leading to loss of antimicrobial activity or formation of potentially irritating by-products [4]. Stability studies must therefore be designed to reflect realistic storage and use conditions and should be correlated with biological endpoints.

Loss of antimicrobial potency during storage undermines both efficacy and safety assumptions derived from early testing. Integration of stability data with antimicrobial and cytotoxicity assessment ensures that the product maintains its intended profile throughout its declared shelf life [6].

### 10.7. Critical Quality Attributes of the Finished Topical Product

Once a candidate enters formulation, the object of evaluation changes from a substance to a product, and the attributes that determine performance change accordingly. Defining critical quality attributes at this point aligns development with established pharmaceutical development principles and makes the link between formulation variables and biological performance explicit rather than empirical [25,84].

For semisolid topical products incorporating natural antimicrobials, the attributes that should be defined, measured and controlled include pH, which affects both stability and the ionization state of phenolic constituents; rheological behaviour, viscosity and spreadability, which govern application and residence time and differ substantially between structurally different commercial systems [85]; globule or droplet size and its distribution in emulsified and nanostructured systems; phase behaviour and physical homogeneity; content of the selected marker constituents, which links the finished product back to the pre-analytical characterization; oxidative status of susceptible constituents; and water activity, which is relevant both to microbiological stability and, for honey-containing products, to the osmotic component of activity. Development work on oil-in-water creams incorporating plant extracts illustrates how these attributes are defined and measured in practice for a semisolid carrying a complex natural matrix [86].

### 10.8. In Vitro Release and Permeation Testing, and the Meaning of Permeation for a Topical Antimicrobial

Product performance testing addresses a question that neither antimicrobial nor cytotoxicity assays answer: whether the active constituents are released from the vehicle and become available at the site where activity is required. The regulatory guidance cited below was developed for the comparative assessment of generic topical products in abbreviated new drug applications and is used here as a methodological reference framework rather than as a requirement directly applicable to the development of a new natural antimicrobial product. In vitro release testing, conducted with a synthetic membrane under standardized conditions, characterizes release from the vehicle and is sensitive to formulation and process changes, which makes it a useful discriminating tool during development and a means of detecting changes between batches [24,87]. In vitro permeation testing, conducted with excised skin in a diffusion cell, characterizes the distribution of the active material into and across the barrier [87,88,89,90].

An interpretive point specific to topical antimicrobials deserves emphasis, because performance criteria transferred uncritically from transdermal development can be misleading. Transdermal products are designed for controlled systemic delivery, whereas locally acting topical products require indication-specific local availability while minimizing unnecessary systemic passage. The target compartment is itself indication-dependent, since some cutaneous infections reside in the stratum corneum while others involve the viable epidermis, the dermis or the follicular unit, and it should be stated explicitly. The desirable profile is therefore sufficient release and retention within the compartment where the infection resides, with systemic absorption limited to what the indication requires, since unnecessary systemic exposure adds no local therapeutic benefit while reintroducing the selective pressure that topical application was chosen to avoid [87,88]. More broadly, the general pharmacological objective of avoiding unnecessary off-target systemic exposure is consistent with observations from other therapeutic contexts in which systemic treatment has been associated with measurable host-level metabolic effects [91]. Permeation data should therefore be interpreted against a target profile defined by the indication, in which retention in the relevant compartment and the ratio of retained to permeated material are the informative parameters, rather than against a maximization criterion.

Antimicrobial activity must then be reconfirmed on the finished formulation rather than inferred from the raw material, since excipients may bind, inactivate or sequester active constituents and alter their partitioning [47,85]. Where release and permeation profiles are correlated with retained antimicrobial activity and with the stability data generated over the intended shelf life, formulation ceases to be a downstream step and becomes an active means of widening the therapeutic window.

### 10.9. Iterative Integration of Formulation and Biological Evaluation

Formulation development should be viewed as an iterative process rather than a linear downstream step. Initial biological data inform formulation choices, while feedback from formulation testing may necessitate adjustment of concentration, vehicle composition, or delivery strategy [6,17]. This cyclical interaction allows progressive optimization of the balance between efficacy, safety, and usability.

By embedding biological evaluation within formulation development, rather than treating it as a separate upstream activity, researchers can more effectively address limitations and exploit opportunities inherent in natural antimicrobial products.

## 11. Resistance Potential, Translational Considerations and Limitations

### 11.1. Potential for Resistance Development and Its Assessment

Since antimicrobial resistance is the principal justification for developing natural topical adjuvants, the framework would be incomplete if it did not address whether the adjuvants themselves can select for resistance. The assumption that multicomponent activity precludes resistance is frequently stated and is not supported by the available evidence.

Exposure of bacteria to sub-inhibitory concentrations of individual essential oil constituents has been shown to select for stable resistant mutants, and sub-inhibitory exposure to essential oils has been associated with reduced susceptibility to conventional antibiotics [78,79]. Cinnamaldehyde induces efflux pump expression and a multidrug-resistant phenotype in *Pseudomonas aeruginosa*, and adaptation of *Salmonella* to linalool has been linked to antibiotic resistance and environmental persistence [80,81]. Development of reduced susceptibility to tea tree oil and its principal constituent has been documented under stepwise selection [82]. Against this, continuous exposure to eugenol and citral did not induce resistance in the species examined, and repeated exposure to medical-grade manuka honey has not selected for resistant variants, which is consistent with the multifactorial and largely physicochemical basis of its activity [77,92]. The picture is therefore neither uniformly reassuring nor uniformly alarming, and it differs between matrix classes.

A defensible formulation of the argument is accordingly the following: multicomponent activity may reduce the probability of single-step target-based resistance, but it does not preclude adaptation, induced tolerance, efflux-mediated responses or cross-resistance to unrelated antimicrobials. This distinction matters particularly for topical products, where concentration gradients at the periphery of the applied area create sub-inhibitory exposure by definition, and where use may be repeated over extended periods.

The framework therefore includes assessment of resistance potential as an advanced evaluation stage for candidates that have passed the earlier gates. Serial passage in the presence of sub-inhibitory concentrations, with determination of the inhibitory concentration at defined intervals and assessment of stability after passage in the absence of the product, provides an interpretable first-line assessment. Susceptibility to clinically relevant antibiotics should be measured before and after exposure, since induction of cross-resistance would be a more serious finding than a modest increase in the inhibitory concentration of the natural product itself [79,80]. Where the intended use is prolonged, absence of such data should be stated as a limitation rather than left to inference.

### 11.2. Regulatory and Translational Considerations

From a translational perspective, increasing emphasis on quality control, traceability, and standardized evaluation aligns natural antimicrobial products more closely with regulatory expectations for topical pharmaceuticals [20]. Early incorporation of these principles reduces uncertainty during later development stages and supports more efficient progression toward clinical evaluation.

A further question concerns the resources the framework presumes. It is modular in execution and sequential in inference. Gates 1 to 5 require standardized microbiology, cell culture and, at Gate 4, access to reconstructed epidermis models, which is the principal resource constraint in that range; the reporting requirements themselves, which constitute much of what the framework asks for, cost documentation rather than equipment. A laboratory that completes Gates 1 to 5 and states where it stopped has produced a valid characterization, and the framework is intended to be usable in that mode. What it maintains is that translational viability is a claim that Gate 6 evidence supports, and not a status conferred by favourable earlier results, since activity is frequently lost or altered on incorporation into a vehicle. Not attempting Gate 6 is a limitation of the study; failing Gate 6 is a finding about the candidate, and conflating the two is one source of the pattern of promising in vitro results that do not translate.

Future progress will depend on continued collaboration between microbiologists, toxicologists, formulation scientists, and regulatory experts to ensure that methodological rigor is maintained while innovation is encouraged.

### 11.3. Strengths and Limitations

The principal strength of this work is integrative rather than empirical. By bringing raw-material standardization, antibacterial and antifungal testing, biofilm endpoints, tiered strain selection, tissue-level safety, selectivity, formulation performance and resistance assessment into a single stage-gated structure, the framework makes explicit a set of decisions that are usually taken implicitly and reported inconsistently. The accompanying reporting checklist and matrix-specific control table are intended for direct use at the bench and do not depend on acceptance of the framework as a whole.

Several limitations should be stated plainly. This is a structured narrative review: the search strategy is described in sufficient detail to be repeated, but source selection was purposive and prioritized methodological relevance, so the evidence base is not an exhaustive representation of the field and is subject to selection by the authors. Although screening, full-text assessment and adjudication were assigned to different authors, the decision to retain a source rested on judgement of methodological coverage rather than on a predefined quantitative rule, and a different team could reasonably have retained a different set. One predefined search, T1, was initially limited to the 1000 most highly cited records from 1310 identified records, a truncation that may have favoured established literature and underrepresented recent but methodologically relevant studies with lower citation counts. This was addressed after the initial submission by re-running the search and exporting the complete result set; screening of the 226 previously unexported records, of which 124 were published in 2025 or 2026, yielded three additional eligible sources, which have been incorporated. The remainder reported the activity of a single matrix without transferable methodological content. Targeted supplementary searches in PubMed/MEDLINE were used for domains in which the primary search provided insufficient topic-specific coverage.

The framework itself has not been validated prospectively. Its gates, the evidence required at each of them and the descriptors applied to selectivity are derived from published methodology and from regulatory principles, not from an interlaboratory study or from a cohort of candidates taken through the full sequence. No threshold values are proposed for the Selectivity Index, and this reflects a genuine absence of consensus rather than a deliberate omission. Similarly, selectivity relative to the resident cutaneous microbiota is introduced as a decision criterion and a reporting requirement, not as a validated parameter, since no accepted index currently exists. Prospective application to defined candidate products, and interlaboratory assessment of the reporting requirements, are the natural next steps and would allow the framework to be refined against practice.

### 11.4. Outlook

In summary, current possibilities in the development of topical natural antimicrobial adjuvants are broader and more robust than at any previous stage. By leveraging integrated evaluation frameworks and advanced formulation strategies, natural products can be repositioned as systematically characterized and methodologically comparable candidates for topical therapy, subject to the prospective validation that this framework has yet to undergo. Future perspectives center on methodological refinement, formulation-driven optimization, and strategic alignment with antimicrobial stewardship principles.

## 12. Conclusions

The evaluation and development of natural products with antimicrobial potential for topical use require an integrated approach that transcends fragmented assessment of biological activity. Minimum inhibitory concentration and minimum bactericidal concentration provide essential information on antimicrobial potency, but their relevance is realized only when interpreted alongside cellular cytotoxicity data and formulation feasibility considerations [2,3,17].

The Selectivity Index emerges as a central decision-making tool, integrating efficacy and safety into a single quantitative parameter applicable at early development stages. Its systematic use supports rational candidate selection, guides formulation strategy, and reduces the risk of advancing products with intrinsic safety limitations [2,6,17].

Go, Conditional Go and No-Go decision criteria translate integrated laboratory data into actionable development pathways, aligning experimental evidence with pharmaceutical and clinical objectives. Through this structured approach, evaluation of natural antimicrobial products becomes a translational process rather than a purely exploratory endeavor.

Pharmaceutical formulation strategies play a decisive role in unlocking the potential of natural antimicrobials, enabling mitigation of limitations related to solubility, stability, and cytotoxicity. Advanced delivery systems and iterative formulation–biology integration further expand the scope of viable candidates.

Overall, the methodological framework presented in this article provides a comprehensive infrastructure for researchers and formulators engaged in the development of topical natural antimicrobial adjuvants. By integrating microbiology, cellular toxicology, and formulation science within a coherent decision-making structure, natural products can progress from empirically investigated substances toward rigorously characterized and translationally viable components of topical therapy.

## Figures and Tables

**Table 1:** Minimum pre-analytical characterization required for each matrix class before entry into quantitative antimicrobial testing.

Domain	Plant Extracts	Essential Oils and Honey
Identity	Species confirmed by an appropriate method; voucher specimen deposited; plant part stated	Essential oils: species and chemotype. Honey: botanical and geographical origin; medical grade or not
Origin and processing	Geographical origin; harvest period; drying and storage conditions	Essential oils: extraction method; storage, headspace and light exposure. Honey: processing and sterilization method
Production parameters	Extraction solvent and concentration; drug-to-extract ratio; extraction yield	Essential oils: yield and time from distillation. Honey: moisture, water activity, pH, sugar profile
Chemical characterization	HPLC or LC-MS fingerprint; quantification of justified marker compounds	Essential oils: GC-MS and GC-FID profile with major constituents quantified; oxidative status. Honey: peroxide versus non-peroxide activity; methylglyoxal where claimed
Reproducibility	Between-batch variability of marker compounds across at least two independent lots	Between-batch variability of major constituents or of antibacterial activity across at least two independent lots
Matched control	Solvent control identical in composition and proportion	Essential oils: emulsifier control at identical final concentration. Honey: artificial honey control matched for sugar composition

**Table 2:** Summary of methods used for the evaluation of antimicrobial activity of natural products and their relevance for formulation.

Method	Experimental Principle	Information Obtained	Major Limitations
Agar diffusion	Diffusion of product in solid medium	Qualitative screening	Diffusibility, volatility
Broth microdilution	Inhibition of growth in liquid medium	Minimum inhibitory concentration	Turbidity, solubility
Bactericidal testing	Subculture on solid agar	Bacterial killing	Reversible effects
Time-kill assay	CFU dynamics over time	Speed and stability of effect	Labor-intensive
Antibiofilm assays	Biomass or metabolic activity	Relevance for chronic infections	Simplified model

**Table 3:** Frequent sources of error and control measures in antimicrobial testing of natural products.

Source of Error	Impact on Results	Control Measure
Biologically active solvent	False-positive activity	Identical solvent control
Intrinsic turbidity	False-negative or false-positive	Subculture, compatible indicators
Essential oil volatility	Overestimation of effect	Closed test systems
High viscosity	Limited diffusion	Adapted dilution strategy

**Table 4:** Matrix-specific assay interferences, required controls and recommended solutions.

Matrix	Principal Assay Problem	Required Control	Recommended Solution
Coloured or turbid extract	Optical interference with turbidimetric or colorimetric reading	Matrix blank at each concentration	Orthogonal viability readout or subculture on solid medium
Viscous extract or honey	Limited diffusion in agar-based assays	Diffusion control with a reference substance	Broth-based quantitative assay rather than diffusion screening
Essential oil	Volatility causing loss or transfer of active material	Vapour-phase control in adjacent wells	Sealed or closed test system; report seal type
Essential oil	Intrinsic activity or toxicity of the emulsifier	Emulsifier control at identical final concentration	Select an emulsifier inactive at the concentration used; report it
Essential oil	Oxidation during storage or testing	Compositional re-analysis at time of testing	Controlled storage; limit interval between analysis and testing
Essential oil, prolonged incubation	Restricted gas exchange under a tight seal suppressing growth of aerobic fungi	Sealed growth control without test product, and unsealed control in parallel	Report seal type, plate format and headspace volume; confirm endpoint viability by subculture
Honey	Osmotic effect indistinguishable from specific activity	Artificial honey control matched for sugars	Report activity relative to the sugar-matched control
Honey	Peroxide-dependent component confounding attribution	Catalase-treated parallel assay	Report peroxide and non-peroxide contributions separately
All matrices	Carry-over into recovery medium during MBC, time-kill or biofilm assays	Neutralization and recovery validation	Validated neutralizer or dilution beyond the inhibitory threshold
All matrices	Adsorption of lipophilic constituents to plasticware	Concentration verification in the test system	Alternative plate material or verified recovery

**Table 5:** Proposed minimum information for reporting antimicrobial testing of natural products intended for topical use.

Domain	Proposed Minimum Information
Natural product identity	Species and, for essential oils, chemotype; plant part; batch; geographical origin; voucher where applicable
Extraction and processing	Solvent and concentration; drug-to-extract ratio; duration; yield; storage conditions
Chemical characterization	Fingerprint method; marker compounds and their quantification; oxidative status where relevant
Microorganisms	Species; strain designation; source and tier (reference, clinical, resistant); year of isolation for clinical isolates
Inoculum	Final concentration; preparation and standardization method; verification procedure
Assay	Method and reference standard followed; growth medium; incubation temperature, atmosphere and duration; readout
Vehicle	Type and final concentration in test wells
Controls	Growth, sterility, vehicle or solvent, positive control, and matrix-specific controls per Table 4
MIC and MBC or MFC	Definition of endpoint used; method; number of technical and biological replicates; values with dispersion
Biofilm	Phenomenon studied; biofilm age; model; readouts used; parameter reported (MBIC, MBEC or percentage reduction)
Cytotoxicity	Cell type and source; exposure duration; assay and endpoint; CC50 with 95% confidence interval
Selectivity Index	Formula used; units of both terms; value reported per microorganism
Topical safety	Model used for irritation and, where applicable, sensitisation and phototoxicity
Formulation	Composition; pH; rheology; marker content; antimicrobial activity reconfirmed on the finished product
Reproducibility	Number of independent biological replicates and of product batches tested
Microbiome selectivity	Commensal species included in the panel; inhibitory concentrations reported alongside those for the target pathogen; commensal sparing ratio reported per pair where the comparison is made
Formulation performance	Critical quality attributes measured; IVRT conditions and membrane; IVPT skin source and conditions; retained versus permeated fractions
Resistance potential	Serial passage design; number of passages; MIC at defined intervals; stability after passage without the product; susceptibility to comparator antibiotics before and after exposure
Translational readiness	Standards and guidelines followed; deviations and their justification; stability data over the intended shelf life; Go, Conditional Go or No-Go outcome and its basis

**Table 6:** Stage-gate criteria of the proposed framework, with the evidence required at each gate and the corresponding Go, Conditional Go and No-Go outcomes. The table represents a qualitative decision architecture rather than a validated set of universal cut-off values; qualifiers such as adequate, acceptable, intermediate and attainable must be defined in advance for each indication, for example within a target product profile or development plan, so that the same data lead to the same decision in different laboratories.

Gate	Minimum Evidence	Go	Conditional Go	No-Go
1. Raw-material identity and standardization	Species and, where applicable, chemotype confirmed; processing documented; fingerprint and markers quantified; two independent batches	Between-batch variability of markers within predefined limits	Variability outside limits but controllable by defined sourcing or processing changes	Identity unconfirmed, or variability not controllable by feasible standardization
2. Quantitative antimicrobial efficacy	MIC and, where relevant, MBC for bacteria or MFC for fungi, by reference broth dilution with matrix-specific and vehicle controls; replicates reported	Reproducible MIC across independent experiments and batches, at concentrations attainable in a topical vehicle	Activity reproducible but attainable only with a solubilization or delivery approach yet to be developed	Activity attributable to vehicle or artefact, or not reproducible
3. Biofilm and clinical-isolate confirmation	Biofilm phenomenon specified and biofilm age stated; at least two complementary readouts addressing different quantities, that is biomass together with a viability or metabolic readout; parameter named as MBIC, MBEC or percentage reduction at a stated concentration; validated neutralization or recovery procedure; tier 2 clinical isolates; tier 3 where AMR relevance is claimed	Activity retained against clinical isolates and against the relevant biofilm phenotype	Activity against planktonic cells only; biofilm or clinical-isolate data pending	Activity lost against clinical isolates or against established biofilm where this is the target
4. Host-tissue safety	Monolayer cytotoxicity with CC50 and confidence intervals; RhE irritation; sensitisation where constituents warrant it	Irritation hazard not identified at the intended use concentration; tolerability for the intended site, duration and barrier status to be confirmed clinically	Irritation potential present but plausibly mitigated by formulation; sensitisation assessment pending	Irritation or sensitisation findings incompatible with the intended use
5. Selectivity	SI reported per microorganism with matched units and exposure; commensal comparison, with the commensal sparing ratio reported per pair, where use is prolonged	Safety margin adequate for the intended indication	Intermediate margin, with a defined formulation strategy to widen it	Efficacy achieved only at or above cytotoxic concentrations
6. Formulation and performance	CQA defined and measured; IVRT; IVPT where local availability matters; antimicrobial activity reconfirmed on the finished product	Activity retained in the finished product with an acceptable release and retention profile	Activity retained but release, retention or physical stability requires optimization	Activity lost on incorporation, or no technologically feasible route identified
7. Resistance and translational readiness	Where prolonged or repeated use is intended, or where claims relating to antimicrobial resistance are made: serial sub-inhibitory passage with MIC monitoring and comparator-antibiotic susceptibility. In all cases: stability over the intended shelf life; documentation and traceability complete	No selection of resistance or cross-resistance under the conditions tested, or assessment not indicated for the intended use; documentation complete	Assessment pending or incomplete; progression conditional on its completion	Selection of stable resistance or of cross-resistance to clinically relevant antibiotics

**Table 7:** Retrospective application of the framework to medical-grade manuka honey. The table reports the class of evidence available at each gate and the outcome it supports under the criteria of Table 6, not a validation of the framework.

Gate	Evidence Available in the Published Literature	Application of the Framework Criteria	Outcome
Gate 1. Raw-material identity and standardization	Botanical origin, medical-grade designation, sterilization by gamma irradiation, standardized antibacterial activity, methylglyoxal content, moisture, pH and sugar profile documented for the medical-grade product [33,50,51]	Characterization set of Table 1 satisfied for the medical-grade product; a retail honey of the same declared floral type would not satisfy it	Go
Gate 2. Quantitative antimicrobial efficacy	Inhibitory concentrations against staphylococci and Gram-negative wound pathogens determined by broth dilution, with the sugar-matched artificial honey control and the catalase-treated parallel assay separating osmotic and peroxide contributions from non-peroxide activity [10,33,50]	Activity reproducible and not attributable to osmolarity alone; concentrations attainable in a topical vehicle, since the matrix is itself the vehicle	Go
Gate 3. Biofilm and clinical-isolate confirmation	Activity reported against established biofilms of wound-relevant species and against contemporary clinical isolates [11,33,61,62]	Phenomenon and biofilm age must be stated and two complementary readouts reported with a validated neutralization step; where biomass staining alone is reported, the gate is not closed	Go, or Conditional Go where the readout requirement is not met
Gate 4. Host-tissue safety	Keratinocyte and fibroblast viability data available; reconstructed human epidermis testing of the finished dressing or formulation rather than of the raw matrix is reported less consistently [51,69]	Illustrates the distinction between monolayer cytotoxicity and topically relevant exposure developed in Section 8.8	Go where reconstructed epidermis data on the finished product are available; otherwise Conditional Go
Gate 5. Selectivity	Selectivity Index calculable per microorganism where inhibitory and cytotoxic concentrations are expressed on the same basis [66]; comparative inhibitory concentrations for resident cutaneous commensals are sparse [73,74]	The gate at which even a well-documented agent is incompletely evidenced, corresponding to the gap identified in Section 8.9	Conditional Go
Gate 6. Formulation and performance	Viscosity, adherence and moisture-donating behaviour characterized; licensed wound-care products exist; critical quality attributes and release behaviour defined for the finished product [11,51]	Permeation interpreted against a retention rather than a maximization target, consistent with Section 10.8	Go
Gate 7. Resistance and translational readiness	Repeated exposure to medical-grade manuka honey has not selected for resistant variants, consistent with a multifactorial and largely physicochemical mode of action [77]	Contrasts with the selection documented for single essential oil constituents under sub-inhibitory exposure [78,79,80,81,82], and shows that Gate 7 discriminates between matrix classes	Go

## Data Availability

The original contributions presented in this study are included in the article and Supplementary Materials. Further inquiries can be directed to the corresponding authors.

## Supplementary Materials

The following supporting information can be downloaded at: https://www.mdpi.com/article/10.3390/pharmaceutics18091111/s1, File S1: Literature search strategy, results and sources retained; File S2: Bibliographic working dataset, including the search log, prioritization worksheet, deduplicated literature dataset and T1 complement screening record.

## Abbreviations

The following abbreviations are used in this manuscript:

CC50	Half-maximal cytotoxic concentration
CFU	Colony-forming unit
CQA	Critical quality attribute
GC-FID	Gas chromatography with flame ionization detection
GC-MS	Gas chromatography-mass spectrometry
HPLC	High-performance liquid chromatography
IVPT	In vitro permeation testing
IVRT	In vitro release testing
LC-MS	Liquid chromatography-mass spectrometry
MBC	Minimum bactericidal concentration
MBEC	Minimum biofilm eradication concentration
MBIC	Minimum biofilm inhibitory concentration
MFC	Minimum fungicidal concentration
MGO	Methylglyoxal
MIC	Minimum inhibitory concentration
QbD	Quality by design
RhE	Reconstructed human epidermis
SI	Selectivity Index
