# Supplementary material for: From Antimicrobial Activity to Topical Translation: An Integrated Framework for Testing, Cytotoxicity Assessment and Formulation of Plant Extracts, Essential Oils and Honey
Source: Pharmaceutics. 2026 Sep 3;18(9):1111. doi: 10.3390/pharmaceutics18091111 (PMC13610726; doi:10.3390/pharmaceutics18091111)
Supplement: Supplementary file 1 [file pharmaceutics-18-01111-s001.zip › Supplementary_File_S1.pdf]

# Supplementary Materials

*From Antimicrobial Activity to Topical Translation: An Integrated Framework for Testing, Cytotoxicity Assessment and Formulation of Plant Extracts, Essential Oils and Honey*

## File S1. Literature search strategy, results and sources retained.

All searches were run on 17 August 2026 and restricted to the period January 2021 to August 2026, to English language, and to the document types Article and Review. In Web of Science the filters applied were PY=(2021-2026) AND LA=(English) AND DT=(Article OR Review); these are omitted from the strings below for readability. Two searches (T3 and T5) were superseded during the process and are reported for completeness: T3 returned an unmanageably broad result set and was replaced by the refined string T3b; T5 contained a construction error, in that the term wound* was placed in the same OR group as the natural-product terms and therefore retrieved records without any natural product, and it was replaced by T5b. Records from T5 were retained only as background context.

### Part A. Primary searches (Web of Science Core Collection)

| ID | Thematic domain | String (Web of Science Core Collection, Topic field) | Identified | Exported |
|---|---|---|---|---|
| T1 | Methodology of antimicrobial testing of natural products (broad) | `TS=(("plant extract*" OR "essential oil*" OR honey) AND (antimicrobial OR antibacterial) AND ("minimum inhibitory concentration" OR "broth microdilution" OR "susceptibility testing") AND (method* OR standardi*))` | 1310 | 1000 |
| T1b | As T1, restricted to topical application | `TS=(("plant extract*" OR "essential oil*" OR honey) AND (antimicrobial OR antibacterial) AND ("minimum inhibitory concentration" OR "broth microdilution" OR "susceptibility testing") AND (topical OR skin OR wound* OR dermal OR cutaneous))` | 199 | 199 |
| T2 | Standardization and characterization of plant extracts | `TS=(("plant extract*" OR "herbal extract*") AND (standardization OR authentication OR fingerprint* OR "marker compound*" OR "batch-to-batch"))` | 373 | 373 |
| T3 | Essential oils (broad; superseded) | `TS=("essential oil*" AND (chemotype OR "GC-MS" OR oxidation OR stability) AND (antimicrobial OR antibacterial))` | 6713 | 0 |
| T3b | Essential oils: chemotype, chemical variability, oxidative status | `TS=("essential oil*" AND (chemotype* OR "chemical variability" OR "oxidation product*" OR autoxidation OR "batch-to-batch" OR standardization) AND (antimicrobial OR antibacterial))` | 504 | 504 |
| T4 | Medical-grade honey | `TS=(honey AND ("medical grade" OR "medical-grade" OR manuka OR methylglyoxal) AND (wound* OR antibacterial OR antimicrobial))` | 391 | 391 |
| T5 | Biofilm (initial string; superseded) | `TS=(biofilm* AND ("minimum biofilm inhibitory concentration" OR MBIC OR MBEC OR "biofilm eradication" OR polymicrobial) AND ("natural product*" OR "plant extract*" OR "essential oil*" OR honey OR wound*))` | 543 | 543 |
| T5b | Antibiofilm activity of natural products (corrected) | `TS=(biofilm* AND ("minimum biofilm inhibitory concentration" OR MBIC OR MBEC OR "biofilm eradication" OR "biofilm inhibition" OR "antibiofilm") AND ("natural product*" OR` | 1822 | 1822 |

| | | | | |
|---|---|---|---|---|
| | | "plant extract*" OR "essential oil*" OR honey OR phytochemical*)) | | |
| T6 | Antifungal susceptibility testing | TS=((Candida OR dermatophyte* OR Trichophyton OR "filamentous fungi") AND ("antifungal susceptibility" OR "broth microdilution") AND ("plant extract*" OR "essential oil*" OR "natural product*" OR honey)) | 198 | 198 |
| T7 | Cytotoxicity and Selectivity Index | TS=(("selectivity index" OR CC50 OR cytotoxicity) AND (keratinocyte* OR fibroblast* OR HaCaT) AND ("plant extract*" OR "essential oil*" OR "natural product*" OR honey)) | 703 | 703 |
| T8 | Reconstructed skin models, irritation and sensitisation | TS=(("reconstructed human epidermis" OR "reconstructed skin" OR "skin irritation" OR "skin sensitization" OR "skin sensitisation") AND (test* OR OECD OR "in vitro")) | 1967 | 1968 |
| T8b | Contact sensitisation to essential oils | TS=("essential oil*" AND (sensitization OR sensitisation OR allergen* OR "contact dermatitis" OR "oxidation product*")) | 248 | 248 |
| T9 | Skin microbiome and commensal preservation | TS=(("skin microbiome" OR "skin microbiota") AND (antimicrobial OR antiseptic* OR "Staphylococcus epidermidis") AND (commensal* OR selectivity OR preservation OR dysbiosis)) | 274 | 274 |
| T10 | In vitro release and permeation testing | TS=(("in vitro release testing" OR IVRT OR "in vitro permeation" OR IVPT OR "Franz diffusion") AND (topical OR semisolid OR cream OR gel OR ointment)) | 689 | 689 |
| T11a | Quality by design and critical quality attributes | TS=(("quality by design" OR "critical quality attribute*") AND (topical OR semisolid OR dermal)) | 241 | 241 |
| T11b | Encapsulation and nanostructured delivery of essential oils | TS=((nanoemulsion* OR nanoencapsulat* OR "lipid nanoparticle*" OR liposome* OR nanocarrier*) AND "essential oil*" AND (antimicrobial OR antibacterial)) | 1712 | 1712 |
| T12 | Resistance development under sub-inhibitory exposure | TS=(("sub-MIC" OR subinhibitory OR "serial passage" OR "resistance development" OR "adaptive resistance" OR tolerance) AND ("essential oil*" OR "plant extract*" OR "natural product*" OR honey)) | 2960 | 2960 |

All non-superseded result sets were exported in full, in successive batches ranked by citation count where necessary, except T1, for which only the first 1000 records ranked by citation count were exported out of 1310 identified. Batched retrieval was used for T5b, T8, T11b and T12; for T8 the two batches overlapped by one record, which is removed at de-duplication, which is why 1968 rows correspond to 1967 identified records. The superseded T3 search was not exported. In total, 13,825 records were exported into the working dataset, and 12,456 remained after de-duplication by digital object identifier and, where absent, by normalized title.

## Part B. Supplementary targeted searches (PubMed/MEDLINE)

Three domains were insufficiently covered by the primary searches and were addressed by targeted supplementary searches. These were sorted by relevance and the first 200 records of each were screened by title and abstract.

| ID | Thematic domain | String (PubMed/MEDLINE, Best Match) | Results | Screened |
|---|---|---|---|---|

| | | | | |
|---|---|---|---|---|
| S-T2 | Authentication and standardization of botanicals | `(plant extract OR botanical OR herbal) AND (authentication OR standardization OR quality control OR fingerprint)` | 47,990 | 200 |
| S-T7 | Application of the Selectivity Index | `"selectivity index" AND (antimicrobial OR antibacterial) AND (cytotoxicity OR keratinocytes OR fibroblasts)` | 582 | 200 |
| S-T12 | Sub-inhibitory exposure and resistance | `(subinhibitory OR sub-MIC OR serial passage) AND (essential oil OR carvacrol OR thymol OR eugenol OR plant extract OR honey) AND (resistance OR adaptation OR tolerance)` | 344 | 200 |

## Part C. Sources retained per thematic domain

Selection was purposive and prioritized methodological relevance for topical natural antimicrobial products over exhaustive coverage, consistent with the structured narrative design of the review. The retained sources are those listed in the reference list of the main manuscript; per-domain counts are given here rather than reference numbers, so that the table remains valid irrespective of the final numbering.

| Thematic domain | Sources retained |
|---|---|
| Review methodology and reporting | 1 |
| Standardization and authentication of plant extracts | 7 |
| Essential oils: chemotype, oxidative status, encapsulation | 2 |
| Honey: grade, mechanisms, testing methodology | 3 |
| Antifungal susceptibility and Candida biofilms | 2 |
| Biofilm endpoints and wound microbiota | 4 |
| Cytotoxicity and Selectivity Index | 3 |
| Skin models, irritation and sensitisation | 3 |
| Skin microbiome and commensal preservation | 3 |
| In vitro release and permeation testing | 3 |
| Quality by design and critical quality attributes | 2 |
| Resistance under sub-inhibitory exposure | 7 |
| Subtotal, sources retained from the predefined update searches | 40 |
| Normative documents retrieved from issuing organizations | 11 |
| Additional peer-reviewed literature identified through hand-searching and contextual supplementation | 38 |
| Total references | 89 |

## Part D. Eligibility criteria

Included: peer-reviewed articles and reviews relevant to at least one thematic domain and to topical application, with methodology described in sufficient detail to be cited as a source of good practice; normative documents in the version currently in force, retrieved from the issuing organization.

Excluded: conference abstracts, preprints and other non-peer-reviewed sources; studies confined to food applications without transferable methodological content; articles for which the full text could not be obtained; retracted articles.

Screening was performed by title and abstract, and retained records were assessed in full text. Priority was given to primary studies for factual statements and to recent reviews for context and synthesis. These eligibility criteria were applied to the predefined and targeted evidence searches; additional peer-reviewed sources identified through hand-searching or contextual supplementation were used selectively to support broader methodological or translational considerations and were not treated as evidence defining the stage-gate criteria.

## Part E. Complementary screening of search T1

Search T1 was originally exported under citation ranking, with the 1000 most highly cited of 1310 identified records retrieved. Following peer review, the search was re-run in the Web of Science Core Collection on 26 August 2026 with the identical string and filters, and the complete result set of 1317 identified records was exported in two batches.

The new export was de-duplicated against the working dataset reported in this supplementary material, by digital object identifier and, where the identifier was absent, by normalized title. This identified 226 records that had not formed part of the original citation-ranked export, of which 124 were published in 2025 or 2026.

All 226 records were screened by title and abstract against the eligibility criteria set out in Part D. Three met them and were incorporated into the revised manuscript: a harmonized broth microdilution protocol for essential oils; a comparative characterization of vapour-phase and liquid-phase composition and antimicrobial activity; and a report of a microplate sealing device developed for vapour-phase susceptibility testing. The remaining 223 records reported the antimicrobial activity of a single plant matrix without transferable methodological content and were excluded on the grounds applied throughout the review.

The complete screening record, listing all 226 records with the screening outcome for each, is provided as the sheet T1 complement screening in Supplementary File S2.
